# Interoperable and scalable data analysis with microservices: applications in metabolomics

**DOI:** 10.1093/bioinformatics/btz160

**Published:** 2019-03-09

**Authors:** Payam Emami Khoonsari, Pablo Moreno, Sven Bergmann, Joachim Burman, Marco Capuccini, Matteo Carone, Marta Cascante, Pedro de Atauri, Carles Foguet, Alejandra N Gonzalez-Beltran, Thomas Hankemeier, Kenneth Haug, Sijin He, Stephanie Herman, David Johnson, Namrata Kale, Anders Larsson, Steffen Neumann, Kristian Peters, Luca Pireddu, Philippe Rocca-Serra, Pierrick Roger, Rico Rueedi, Christoph Ruttkies, Noureddin Sadawi, Reza M Salek, Susanna-Assunta Sansone, Daniel Schober, Vitaly Selivanov, Etienne A Thévenot, Michael van Vliet, Gianluigi Zanetti, Christoph Steinbeck, Kim Kultima, Ola Spjuth

**Affiliations:** 1 Department of Medical Sciences, Clinical Chemistry, Uppsala University, Uppsala, Sweden; 2 European Molecular Biology Laboratory, European Bioinformatics Institute (EMBL-EBI), Cambridge, UK; 3 Department of Computational Biology, University of Lausanne, Lausanne, Switzerland; 4 Swiss Institute of Bioinformatics, Lausanne, Switzerland; 5 Department of Neuroscience, Uppsala University, Uppsala, Sweden; 6 Department of Information Technology, Uppsala University, Uppsala, Sweden; 7 Department of Pharmaceutical Biosciences, Uppsala University, Uppsala, Sweden; 8 Department of Biochemistry and Molecular Biomedicine, and Institute of Biomedicine (IBUB), Faculty of Biology, Universitat de Barcelona (IBUB), Barcelona, Spain; 9 Centro de Investigación Biomédica en Red de Enfermedades Hepáticas y Digestivas (CIBEREHD) and Metabolomics Node at INB-Bioinfarmatics Platform, Instituto de Salud Carlos III (ISCIII), Madrid, Spain; 10 Oxford e-Research Centre, Department of Engineering Science, University of Oxford, Oxford, UK; 11 Division of Analytical Biosciences, Leiden Academic Centre for Drug Research, Leiden University, Leiden, The Netherlands; 12 National Bioinformatics Infrastructure Sweden, Uppsala University, Uppsala, Sweden; 13 Department of Stress and Developmental Biology, Leibniz Institute of Plant Biochemistry, Halle, Germany; 14 German Centre for Integrative Biodiversity Research (iDiv), Halle-Jena-Leipzig, Germany; 15 CRS4: Center for Advanced Studies, Research and Development in Sardinia, Distributed Computing Group, Pula, Italy; 16 CEA, LIST, Laboratory for Data Analysis and Systems' Intelligence, MetaboHUB, Gif-sur-Yvette, France; 17 Faculty of Medicine, Department of Surgery & Cancer, Imperial College London, London, UK; 18 International Agency for Research on Cancer, 69372 Lyon CEDEX 08, France; 19 Institute for Inorganic and Analytical Chemistry, Friedrich-Schiller-University, Jena, Germany

## Abstract

**Motivation:**

Developing a robust and performant data analysis workflow that integrates all necessary components whilst still being able to scale over multiple compute nodes is a challenging task. We introduce a generic method based on the microservice architecture, where software tools are encapsulated as Docker containers that can be connected into scientific workflows and executed using the Kubernetes container orchestrator.

**Results:**

We developed a Virtual Research Environment (VRE) which facilitates rapid integration of new tools and developing scalable and interoperable workflows for performing metabolomics data analysis. The environment can be launched on-demand on cloud resources and desktop computers. IT-expertise requirements on the user side are kept to a minimum, and workflows can be re-used effortlessly by any novice user. We validate our method in the field of metabolomics on two mass spectrometry, one nuclear magnetic resonance spectroscopy and one fluxomics study. We showed that the method scales dynamically with increasing availability of computational resources. We demonstrated that the method facilitates interoperability using integration of the major software suites resulting in a turn-key workflow encompassing all steps for mass-spectrometry-based metabolomics including preprocessing, statistics and identification. Microservices is a generic methodology that can serve any scientific discipline and opens up for new types of large-scale integrative science.

**Availability and implementation:**

The PhenoMeNal consortium maintains a web portal (https://portal.phenomenal-h2020.eu) providing a GUI for launching the Virtual Research Environment. The GitHub repository https://github.com/phnmnl/ hosts the source code of all projects.

**Supplementary information:**

Supplementary data are available at *Bioinformatics* online.

## 1 Introduction

Biology is becoming data-intensive as high throughput experiments in genomics or metabolomics are rapidly generating datasets of massive volume and complexity (Marx, 2013; Schadt *et al.*, 2010), posing a fundamental challenge on large scale data analytics.

Currently, the most common large-scale computational infrastructures in science are shared High-Performance Computing (HPC) systems. Such systems are usually designed primarily to support computationally intensive batch jobs—e.g. for the simulation of physical processes—and are managed by specialized system administrators. This model leads to rigid constraints on the way these resources can be used. For instance, the installation of software must undergo approval and may be restricted, which contrasts with the needs in the analysis where a multitude of software components of various versions—and their dependencies—are needed, and where these need to be continuously updated.

Cloud computing offers a compelling alternative to shared HPC systems, with the possibility to instantiate and configure on-demand resources such as virtual computers, networks and storage, together with operating systems and software tools. Users only pay for the time the virtual resources are used, and when they are no longer needed they can be released and incur no further costs for usage or ownership. For scientists, this constitutes a shift from owning computer hardware, to starting up Infrastructure-as-a-Service (IaaS) nodes with virtual machines on cloud resources, with the explicit need to then install all necessary software for the analysis which in many cases constitutes a demanding and time-consuming task (Langmead and Nellore, 2018). Along with infrastructure provisioning, software provisioning—i.e. installing and configuring software for users—has also advanced. Consider, for instance, containerization (Silver, 2017), which allows entire applications with their dependencies to be packaged, shipped and run on a computer but isolated from one another in a way analogous to virtual machines, yet much more efficiently. Containers are more compact, and since they share the same operating system kernel, they are fast to start and stop and incur little overhead in execution. These traits make them an ideal solution to implement lightweight *microservices*, a software engineering methodology in which complex applications are divided into a collection of smaller, loosely coupled components that communicate over a network (Newman, 2015). Microservices share many properties with traditional always-on web services found on the Internet, but microservices are generally smaller, portable and can be started on-demand within a separate computing environment. Another important feature of microservices is that they have a technology-agnostic communication protocol, and hence can serve as building blocks that can be combined and reused in multiple ways (da Veiga Leprevost *et al.*, 2017).

Microservices are highly suitable to run in elastic cloud environments that can dynamically grow or shrink on demand, enabling applications to be scaled-up by simply starting multiple parallel instances of the same service. However, to achieve effective scalability a system needs to be appropriately sectioned into microservice components and the data to be exchanged between the microservices needs to be defined for maximum efficiency—both being challenging tasks.

One of the omics fields that faces challenges by data growth is metabolomics which measures the occurrence, concentrations and changes of small molecules (metabolites) in organisms, organs, tissues, cells and cellular compartments. Metabolite abundances are assayed in the context of environmental or dietary changes, disease or other conditions (Nicholson and Wilson, 2003). Metabolomics is, as most other omics technologies, characterized by the use of high-throughput experiments performed using a variety of spectroscopic methods such as Mass Spectrometry (MS) and Nuclear Magnetic Resonance (NMR) that produce large amounts of data (Montenegro-Burke *et al.*, 2017). With increasing data size and number of samples, the analysis process becomes intractable for desktop computers due to requirements on compute cores, memory, storage, etc. As a result, large-scale computing infrastructures have become important components in scientific projects (Liew *et al.*, 2016). Moreover, making use of such complex computing resources in an analysis workflow presents its own challenges, including achieving efficient job parallelism and scheduling as well as error handling (Suplatov *et al.*, 2016). In addition, configuring the necessary software tools and chaining them together into a complete re-runnable analysis workflow commonly requires substantial IT-expertise, while creating portable and fault-tolerant workflows with a robust audit trail is even more difficult. Metabolomics has already benefited from cloud-based systems enabling the users certain preprocessing and main downstream analysis on e.g. MS data. Examples of such systems are XCMS ONLINE (Warth *et al.*, 2017), MetaboAnalyst (Xia *et al.*, 2012), Chorus (chorusproject.org) and The Metabolomics Workbench (Sud *et al.*, 2016) (www.metabolomicsworkbench.org) which provide tools that scale with computational demands.

In this manuscript, we present a method that uses components for data analysis encapsulated as microservices and connected into computational workflows to provide complete, ready-to-run, reproducible data analysis solutions that can be easily deployed on desktop computers as well as public and private clouds. Our work contrasts to previously reported research environments, sometimes termed Virtual Research Environments (Allan, 2009; Candela *et al.*, 2013), Scientific Gateways (Lawrence *et al.*, 2015) and Virtual Labs (Waldrop, 2013), in that it encompasses the complete setup of the computational infrastructure and frameworks to run analysis in a wide range of environments; however our approach requires virtually no involvement in the setup and no special IT skills from the user. The methodology provides a framework for rapid and efficient integration of new tools and developing scalable, and interoperable workflows, supporting multiple workflow engines such as Galaxy (Goecks *et al.*, 2010) and Luigi (https://github.com/spotify/luigi). We validate the method on four metabolomics studies and show that it enables scalable and interoperable data analysis.

## 2 Materials and methods

### 2.1 Microservices

A detailed description of the methods is present in Supplementary Method S1. Briefly, in order to construct a microservice architecture for metabolomics we used Docker (Merkel, 2014) (https://www.docker.com/) containers to encapsulate software tools. Tools are developed as open source and are available in a public repository such as GitHub (https://github.com/), and the PhenoMeNal project containers are built and tested on a Jenkins continuous integration (CI) server (http://phenomenal-h2020.eu/jenkins/). Containers are assembled in different branches using the git versioning system. Builds originating from the development branch of each container repository give rise to container images tagged as ‘development’; builds coming from the master branches result in release images. In order for a container be pushed to the container registry, it must pass a testing criteria which is defined by the developer of the tool. All published containers are thus available for download and can be used in any microservice architecture. Data is exchanged between services by passing references to a shared local file system. The CI system constitutes a key part of the methodology, as it ensures that containers are continuously successfully packaged, versioned, tested and that adequate reporting measures are in place to handle any errors in this process over time.

### 2.2 Virtual Research Environment (VRE)

We developed a Virtual Research Environment (VRE) which uses Kubernetes (https://kubernetes.io/) for orchestration of the containers, including initialization and scaling of jobs based on containers, abstractions to file system access for running containers, exposure of services, as well as rescheduling of failed jobs and long running services. Kubernetes was chosen over other frameworks such as Docker Swarm because of its larger momentum and that it is more widely used in production environments. Docker also provides Kubernetes as part of their Enterprise solutions (and even now the community ones). To enable convenient instantiation of a complete virtual infrastructure, we developed KubeNow (https://github.com/kubenow/KubeNow) (Capuccini *et al.*, 2018) which includes instantiation of compute nodes, shared file system storage, networks, configure DNS, operating system, container implementation and orchestration tools, including Kubernetes, on a local computer or server. In order to deploy applications, we used two main classes of services: long-lasting services, and compute jobs. Long-lasting services were used for applications such as the user interface whereas compute jobs were used to perform temporary functions in data processing. The VRE includes Galaxy, Luigi workflow engine and Jupyter notebook as user-facing services. In the PhenoMeNal CI system, the VRE is instantiated and tested on all supported cloud providers nightly in order to ensure a working system over time.

### 2.3 Demonstrators

We validated our method in the field of metabolomics using four demonstrators. Demonstrators 1 and 2 showcase scalability and interoperability of our microservice-based architecture whereas Demonstrators 3 and 4 exemplify flexibility to account for new application domains, showing the architecture is domain-agnostic.

***Demonstrator 1: Scalability of microservices in a cloud environment.***The objective of this analysis was to demonstrate the computational scalability of an existing workflow on a large dataset [Metabolomics data have been deposited to the EMBL-EBI MetaboLights database (Haug *et al.*, 2013) with the identifier MTBLS233 (Ranninger *et al.*, 2016). The complete dataset can be accessed here https://www.ebi.ac.uk/metabolights/MTBLS233]. The experiment includes 528 mass spectrometry samples from whole cell lysates of human renal proximal tubule cells that were pre-processed through a five-step workflow (consisting of peak picking, feature finding, linking, file filtering and exporting) using the OpenMS software (Sturm *et al.*, 2008). This preprocessing workflow was reimplemented using Docker containers and run using the Luigi workflow engine. Scalability of concurrent running tools (on 40 Luigi workers, each worker receives tasks from the scheduler and executes them) was measured using weak scaling efficiency (WSE), where the workload assigned to each worker stays constant and additional workers are used to solve a larger total problem.

***Demonstrator 2: Interoperability of microservices.***The objective of this analysis was to demonstrate interoperability as well as to present a real-world scenario in which patients’ data are processed using a microservices-based platform. We used a dataset consisting of 37 clinical cerebrospinal fluid (CSF) samples including thirteen relapsing-remitting multiple sclerosis (RRMS) patients and 14 secondary progressive multiple sclerosis (SPMS) patients as well as 10 non-multiple sclerosis controls. 26 quality controls (19 blank and 7 dilution series samples) were also added to the experiment. In addition, 8 pooled CSF samples containing MS/MS data were included in the experiment for improving identification [Metabolomics data have been deposited to the EMBL-EBI MetaboLights database with the identifier MTBLS558. The complete dataset can be accessed here https://www.ebi.ac.uk/metabolights/MTBLS558]. The samples were processed and analyzed on the Galaxy platform (Goecks *et al.*, 2010), running in a VRE behind the Uppsala University Hospital firewall to be compliant with local ELSI (Ethics, Legal, Social implications) regulations.

***Demonstrator 3: 1 D NMR-analysis workflow.***The purpose of this demonstrator was to highlight the fact that the microservice architecture is indeed domain-agnostic and is not limited to a particular assay technology. This NMR-based metabolomics study was originally performed by Salek *et al.* (2007) on urine of type 2 diabetes mellitus (T2DM) patients and controls [Metabolomics data have been deposited to the EMBL-EBI MetaboLights database with the identifier MTBLS1. The complete dataset can be accessed here https://www.ebi.ac.uk/metabolights/MTBLS1]. In total, 132 samples (48 T2DM and 84 controls) were processed using a Galaxy workflow performing conversion, preprocessing, multivariate data analysis and result visualization.

***Demonstrator 4: Start-to-end fluxomics workflow.***The purpose of this demonstrator was to show the integrated use of separately developed tools covering subsequent steps of the study of metabolic fluxes based on ^13^C stable isotope-resolved metabolomics (SIRM) (Buescher *et al.*, 2015; King *et al.*, 2015; Niedenführ *et al.*, 2015). Here we implemented the analysis of flux distributions in HUVEC cells under hypoxia [Metabolomics data have been deposited to the EMBL-EBI MetaboLights database with the identifier MTBLS412. The complete dataset can be accessed here https://www.ebi.ac.uk/metabolights/MTBLS412], from raw mass spectra contained in netCDF files, using a workflow implemented in Galaxy including reading and extraction of the data, correcting the evaluated mass spectra for natural isotopes and computing steady-state distribution of ^13^C label as function of steady-state flux distribution.

### 2.4 Availability and implementation

The PhenoMeNal consortium maintains a web portal (https://portal.phenomenal-h2020.eu) providing a GUI for launching VREs using KubeNow (Capuccini *et al.*, 2018) on a selection of the largest public cloud providers, including Amazon Web Services, Microsoft Azure and Google Cloud Platform, or on private OpenStack-based installations. The Wiki containing documentation is also hosted on GitHub https://github.com/phnmnl/phenomenal-h2020/wiki. The PhenoMeNal Portal can be reached at https://portal.phenomenal-h2020.eu. The public instance of Galaxy is accessible at https://public.phenomenal-h2020.eu. The containers provisioned by PhenoMeNal comprise tools built as open source software that are available in a public repository such as GitHub, and are subject to continuous integration testing. The containers that satisfy testing criteria are pushed to a public container repository, and containers that are included in stable VRE releases are also pushed to Biocontainers (da Veiga Leprevost *et al.*, 2017). The GitHub repository https://github.com/phnmnl/hosts the source code of all development projects. Source code and documentation are available under the terms of the Apache 2.0 license. Integrated open source projects are available under the respective licensing terms. The Demonstrators can be obtained from: Demonstrator 1: https://github.com/phnmnl/MTBLS233-Jupyter; Demonstrator 2: https://public.phenomenal-h2020.eu/u/phenoadmin/w/metabolomics-lcmsms-processing-quantification-annotation-identification-and-statistics-1; Demonstrator 3: https://public.phenomenal-h2020.eu/u/phenoadmin/w/metabolomics-nmr-rnmr1d-metabolights-data-processing-and-plot; Demonstrator 4: https://public.phenomenal-h2020.eu/u/phenoadmin/w/fluxomics-stationary-13c-ms-iso2flux-with-visualization

## 3 Results

**Fig. 1.**
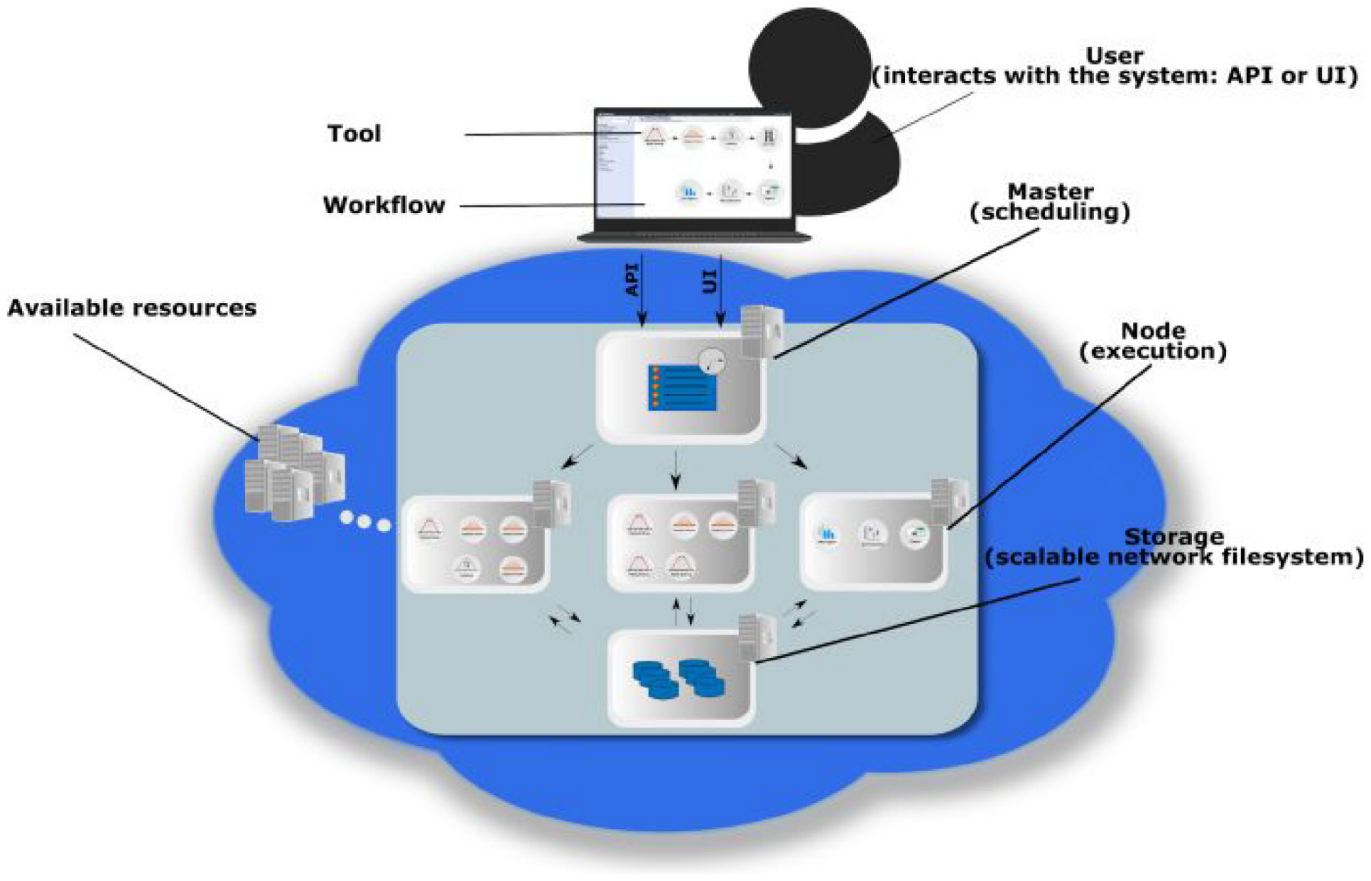
Overview of the components in a microservices-based framework. Complex applications are divided into smaller, focused and well-defined (micro-) services. These services are independently deployable and can communicate with each other, which allows to couple them into complex task pipelines, i.e. data processing workflows. The user can interact with the framework programmatically via an Application Program Interface (API) or via a graphical user interface (GUI) to construct or run workflows of different services, which are executed independently. Multiple instances of services can be launched to execute tasks in parallel, which effectively can be used to scale analysis over multiple compute nodes. When run in an elastic cloud environment, virtual resources can be added or removed depending on the computational requirements

We developed a VRE based on a microservices architecture encapsulating a large suite of software tools for performing metabolomics data analysis (see Supplementary Table S1). Scientists can interact with the microservices programmatically via an Application Programming Interface (API) or via a web-based graphical user interface (GUI), as illustrated in Figure 1. To connect microservices into computational workflows, the two frameworks Galaxy (Goecks *et al.*, 2010) and Luigi (https://github.com/spotify/luigi) were adapted to execute jobs on Kubernetes. Galaxy is a web-based interface for individual tools and allows users to share workflows, analysis histories and result datasets. Luigi on the other hand focuses on scheduled execution, monitoring, visualization and the implicit dependency resolution of tasks (Leipzig, 2017). These basic infrastructure services, together with the Jupyter notebook (Kluyver *et al.*, 2016) interactive programming environment, are deployed as long-running services in the VRE, whereas the other analysis tools are deployed as transient compute jobs to be used on-demand. System and client applications were developed for launching the VRE on desktop computers, public and private cloud providers, automating all steps required to instantiate the virtual infrastructures.

**Fig. 2.**
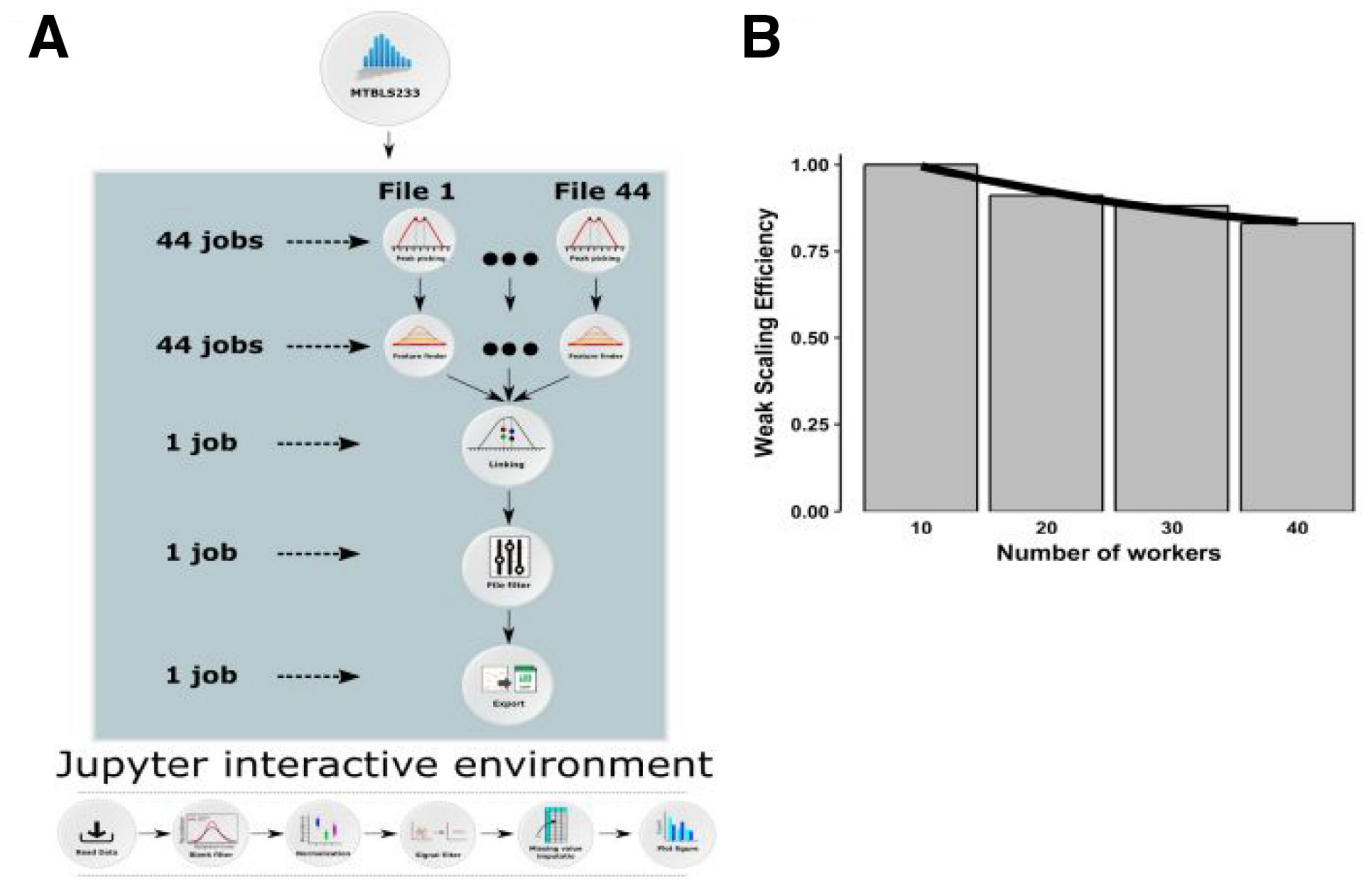
Diagram of scalability-testing on a metabolomics dataset (MetaboLights ID: MTBLS233) in Demonstrator 1 to illustrate the scalability of a microservice approach. A) The preprocessing workflow is composed of 5 OpenMS tasks that were run in parallel over the 12 groups in the dataset using the Luigi workflow system. The first two tasks, peak picking (528 tasks) and feature finding (528 tasks), are trivially parallelizable, hence they were run concurrently for each sample. The subsequent feature linking task needs to process all of the samples in a group at the same time, therefore 12 of these tasks were run in parallel. In order to maximize the parallelism, each feature linker container (microservice) was run on 2 CPUs. Feature linking produces a single file for each group, that can be processed independently by the last two tasks: file filter (12 tasks) and text exporter (12 tasks), resulting in total of 1092 tasks. The downstream analysis consisted of 6 tasks that were carried out in a Jupyter Notebook. Briefly, the output of preprocessing steps was imported into R and the unstable signals were filtered out. The missing values were imputed and the resulting number of features were plotted. B) The weak scaling efficiency plot for Demonstrator 1. Given the full MTBLS233 dataset, the preprocessing was run on 40 Luigi workers. Then for 1/4, 2/4, 3/4 of MTBLS233, the analysis was run again on 10, 20 and 30 workers respectively. For each run, we measured the processing time T10, T20, T30 and T40, and we computed the WSEn = T10/Tn for n = 10, 20, 30, 40. The WSE plot shows scalability up to 40 CPUs, where we achieved ∼88% scaling efficiency. The running time for the full dataset (a total of 1092 tasks) on 40 workers was ∼4 hours

***Demonstrator 1: Scalability of microservices in a cloud environment***. The Diagram of scalability-testing on the metabolomics dataset is illustrated in Figure 2. The analysis resulted to WSE of 88% with an execution time of approximately four hours (online methods, Supplementary Fig. S2), compared with the ideal case of 100% where linear scaling is achieved if the run time stays constant while the workload is increased. In addition, the final result of the workflow (online methods, Supplementary Fig. S3) was identical to that presented by the original MTBLS233 study (Ranninger *et al.*, 2016) in negative ionization mode. However, in the positive ionization mode, one *m*/*z* feature was found in a different group (*m*/*z* range 400–1000) than it was originally reported by Ranninger *et al.* (*m*/*z* range 200–400).

**Fig. 3.**
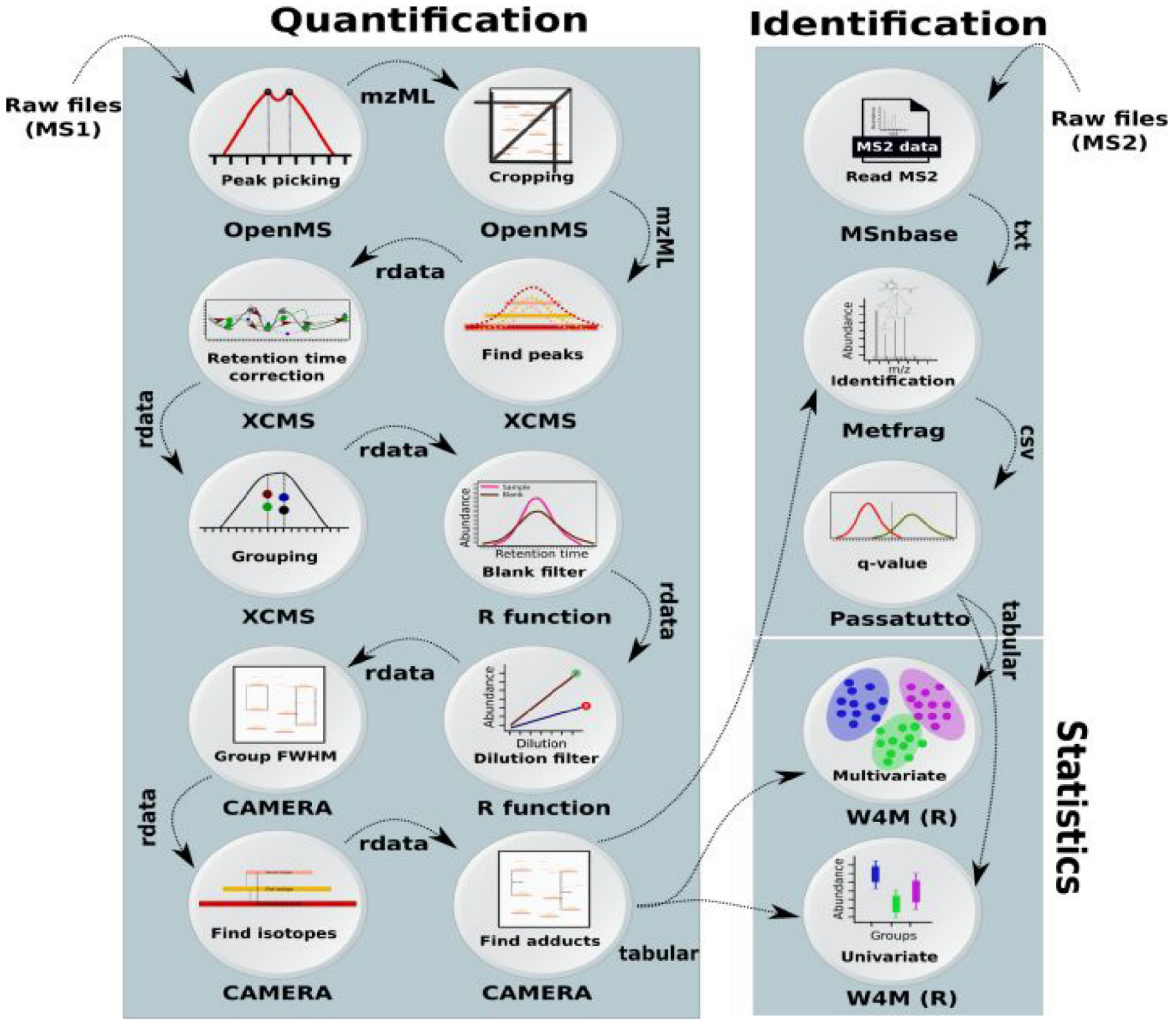
Overview of the workflow used to process multiple-sclerosis samples in Demonstrator 2, where a workflow was composed of the microservices using the Galaxy system. The data was centroided and limited to a specific mass over charge (*m*/*z*) range using OpenMS tools. The mass traces quantification and retention time correction was done via XCMS (Smith *et al.*, 2006). Unstable signals were filtered out based on the blank and dilution series samples using an in-house function (implemented in R). Annotation of the peaks was performed using CAMERA (Kuhl *et al.*, 2012). To perform the metabolite identification, the tandem spectra from the MS/MS samples in mzML format were extracted using MSnbase and passed to MetFrag. The MetFrag scores were converted to q-values using Passatutto software. The result of identification and quantification were used in ‘Multivariate’ and ‘Univariate’ containers from Workflow4Metabolomics (Giacomoni *et al.*, 2015) to perform Partial Least Squares Discriminant Analysis (PLS-DA)

**Fig. 4.**
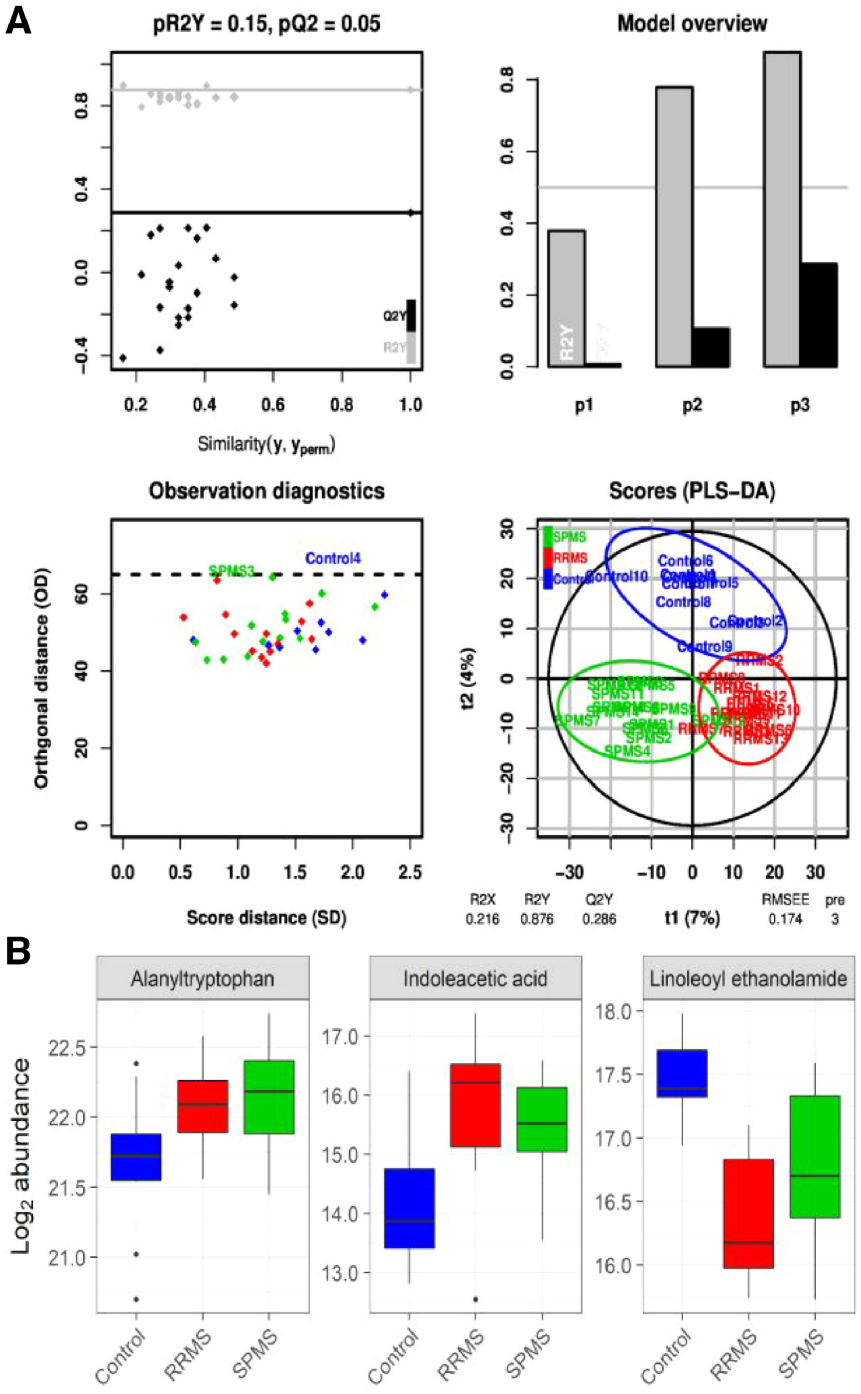
The results from analysis of multiple sclerosis data in Demonstrator 2, presenting new scientifically useful biomedical knowledge. **A**) The PLS-DA results suggest that the metabolite distribution in the RRMS and SPMS samples are different to controls. **B**) Three metabolites were identified as differentially regulated between multiple sclerosis subtypes and control samples, namely Alanyltryptophan and Indoleacetic acid with higher and Linoleoyl ethanolamide with lower abundance in both RRMS and SPMS compared to controls. Abbr., RRMS: relapsing-remitting multiple sclerosis, SPMS: secondary progressive multiple sclerosis

***Demonstrator 2: Interoperability of microservices***. We developed a start to end workflow for pre-processing and statistical analysis of LC-MS metabolomics data (Fig. 3). The workflow allows seamless integration of six major metabolomics data analysis components (26 steps) each was already implemented in independent software suites: noise reduction and filtering [OpenMS (Rost *et al.*, 2016)], quantification, alignment and matching [XCMS (Smith *et al.*, 2006)], filtering of biological non-relevant signals (R), annotation of signals [CAMERA (Kuhl *et al.*, 2012)], identification [MetFrag (Wolf *et al.*, 2010)], statistics [Workflow4Metabolomics (Giacomoni *et al.*, 2015)]. The result of the workflow (multivariate analysis) showed a clear difference in the metabolic constitution between the three disease groups of RRMS, SPMS and non-multiple sclerosis controls (Fig. 4A). In addition, the univariate analysis resulted in a total of three metabolites being significantly altered (p < 0.05) between multiple sclerosis subtypes and control samples, namely alanyltryptophan and indoleacetic acid with higher and linoleoyl ethanolamide with lower abundance in both RRMS and SPMS compared to controls (Fig. 4B).

**Fig. 5.**
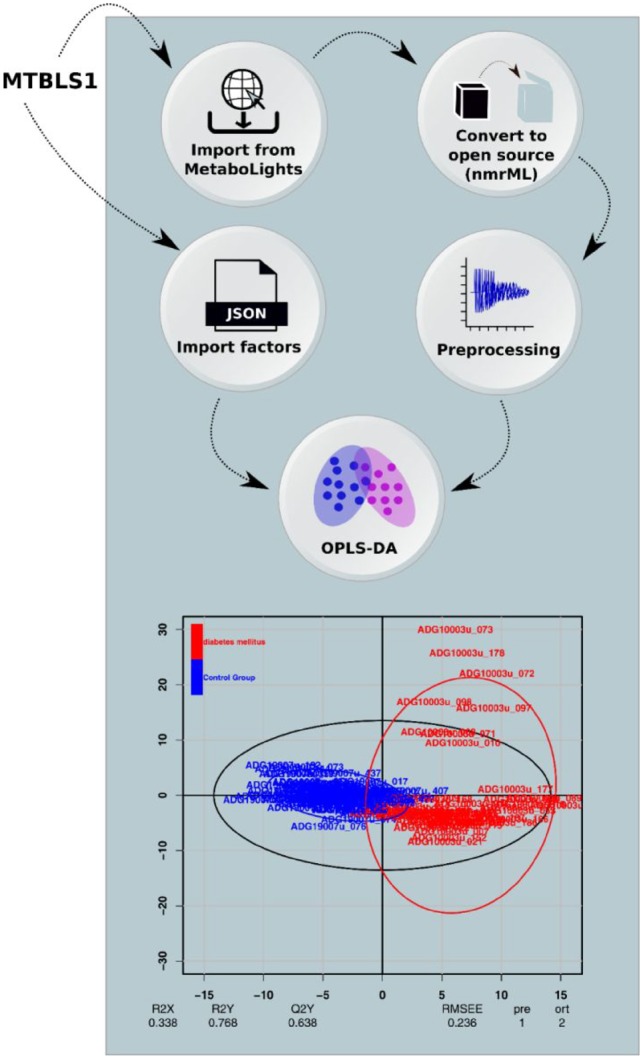
Overview of the NMR workflow in Demonstrator 3. The raw NMR data and experimental metadata (ISATab) was automatically imported from the Metabolights database and converted to open source nmrML format. The preprocessing was performed using the rnmr1d package part of nmrprocflow tools. All study factors were imported from MetaboLights and were fed to the multivariate node to perform an OPLS-DA

**Fig. 6.**
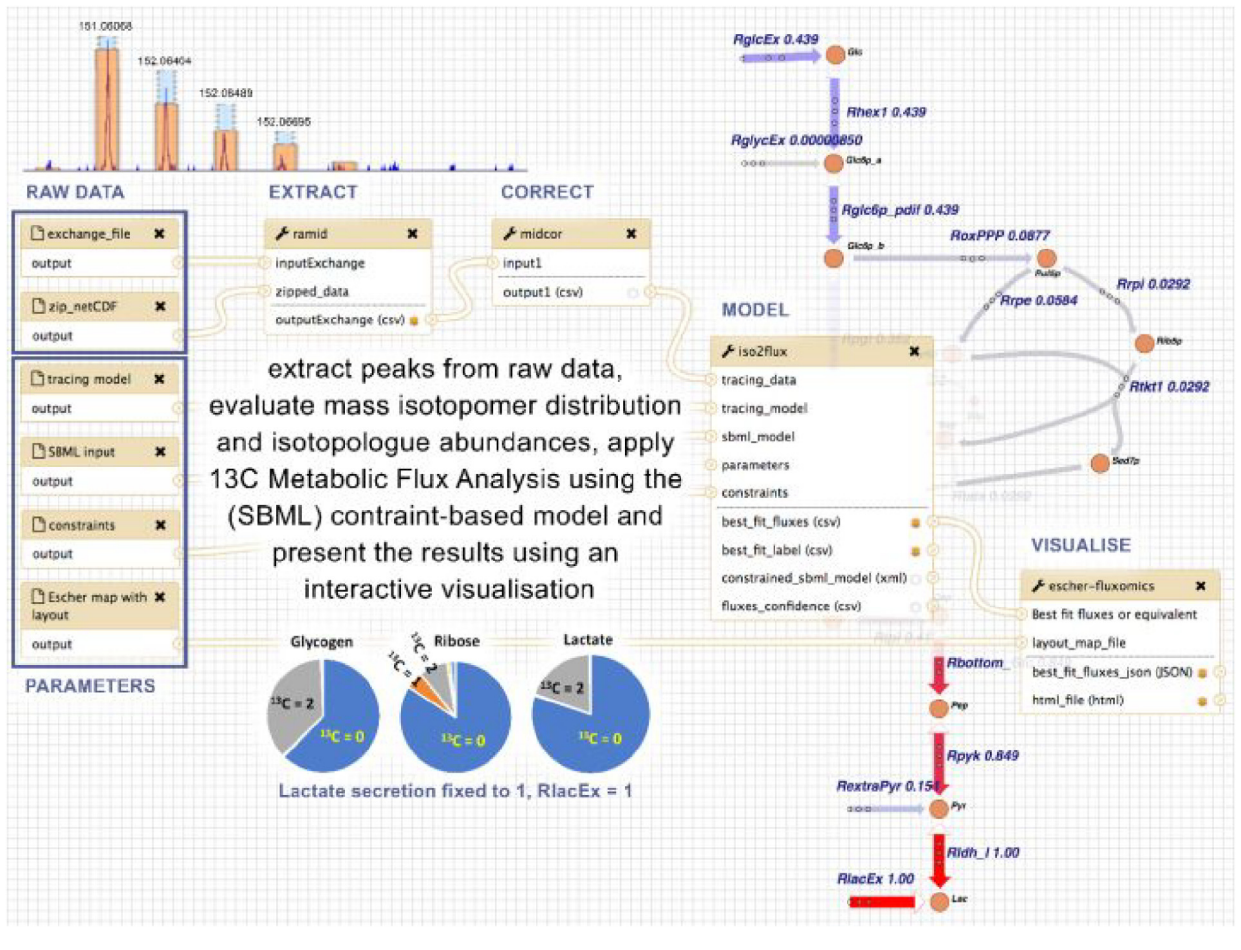
Overview of the workflow for fluxomics, with Ramid, Midcor, Iso2Flux and Escher-fluxomics tools supporting subsequent steps of the analysis. The example refers to HUVEC cells incubated in the presence of [1,2-^13^C_2_]glucose and label (^13^C) propagation to glycogen, RNA ribose and lactate measured by mass spectrometry. Ramid reads the raw netCDF files, corrects baseline and extracts the peak intensities. The resulting peak intensities are corrected (natural abundance, overlapping peaks) by Midcor, which provides isotopologue abundances. Isotopologue abundances, together with a model description (SBML model, tracing data, constraints), are used by Iso2Flux to provide flux distributions through glycolysis and pentose-phosphate pathways, which are shown as numerical values associated to a metabolic scheme of the model by the Escher-fluxomics tool

***Demonstrators 3 and 4: Domain agnosticity (NMR and fluxomics workflows)***. We developed a workflow for analysis of 1 D NMR data. The workflow consisted of automatic downloading NMR vendor data (and metadata) from MetaboLights database followed by format standardization, spectral processing and statistical analysis. We processed a NMR dataset (demonstrator 3) resulting to quantification of a total of 726 features which were used to perform Orthogonal Projections to Latent Structures Discriminant Analysis (OPLS-DA). This resulted in a clear separation between T2DM and controls (Fig. 5), similar to that of previous findings (Salek *et al.*, 2007). Lastly, we designed a workflow for analyzing metabolite metabolic fluxes. The workflow integrated four main steps including data extraction, data correction, calculation of flux distribution and visualization. Using this workflow (Fig. 6), we achieved detailed description of the magnitudes of the fluxes through the reactions accounting for glycolysis and pentose phosphate pathway.

## 4 Discussion

Implementing the different tools and processing steps of a data analysis workflow as separate services that are made available over a network was in the spotlight in the early 2000s (Foster, 2005) as service-oriented architectures (SOA) in science. At that time, web services were commonly deployed on physical hardware and exposed and consumed publicly over the internet. However, it soon became evident that this architecture did not fulfill its promises as it was hard to scale from a computational and maintainability perspective. In addition, the web services were not portable and mirroring them was complicated (if at all possible). Furthermore, API changes and frequent services outage made it frustrating to connect them into functioning computational workflows. Ultimately, the ability to replicate an analysis on local and remote hardware (such as a computer cluster) was very difficult due to heterogeneity in the computing environments.

At first sight microservices might seem similar to above mentioned SOA web services, but microservices can with great benefit be executed in virtual environments (abstracting over OS and hardware architectures) in such a way that they are only instantiated and executed on-demand, and then terminated when they are no longer needed. This makes such virtual environments inherently portable and they can be launched on demand on different platforms (e.g. a laptop, a powerful physical server or an elastic cloud environment). A key aspect is that workflows of microservices are still executed identically, agnostic of the underlying hardware platform. Container-based microservices provide a wide flexibility in terms of versioning, allowing the execution of newer and older versions of each container as needed for reproducibility. Since all software dependencies are encompassed within the container, which is versioned, the risk of workflow failure due to API changes is minimized. An orchestration framework such as Kubernetes further allows for managing errors in execution and transparently handles the restarting of services. Hence, technology has caught up with service-oriented science, and microservices have taken the methodology to the next level, alleviating many of the previous problems related to scalability, portability and interoperability of software tools. This is advantageous in the context of omics analysis, which produces multidimensional datasets reaching beyond gigabytes, on into terabytes, leading to ever-increasing demand on processing performance (Marx, 2013; Schadt *et al.*, 2010). However, containerization does not address how services communicate with each other, but this has to be implemented inside the container itself. Traditional web services addressed this by standardizing the messaging protocol and public-facing interfaces (e.g. SOAP and WSDL) (Stockinger *et al.*, 2008), while in a containerized environment Representational State Transfer (REST) (Fielding, 2000) or passing files by reference to a shared file system is more common. In Demonstrator 1, we showed that microservices enable highly efficient and scalable data analyses by executing individual modules in parallel, and that they effectively harmonize with on-demand elasticity of the cloud computing paradigm. The reached scaling efficiency of ∼88% indicates remarkable performance achieved on generic cloud providers. Furthermore, although our results in positive ionization model was slightly different to that of Ranninger *et al.* (2016), the results of our analysis were replicable regardless of the platform used to perform the computations.

In addition to the fundamental demand for high performance, the increased throughput and complexity of omics experiments has led to a large number of sophisticated computational tools (Berger *et al.*, 2013), which in turn necessitates integrative workflow engines (Atkinson *et al.*, 2017; Di Tommaso *et al.*, 2017; Liew *et al.*, 2016). In order to integrate new tools in such workflow engines, compatibility of the target environment, tools and APIs needs to be considered (Di Tommaso *et al.*, 2017). Containerization facilitates this by providing a platform-independent virtual environment for developing and running the individual tools. However, the problem of compatibility between tools/APIs and data formats remains and needs to be tackled by international consortia (Wilkinson *et al.*, 2016). Our methodology the currently non-trivial task of instantiating the complete microservice environment through a web portal that allows for convenient deployment of the VRE on public cloud providers. Moreover, using this web portal, microservices and VREs can be deployed on a trusted private cloud instance or a local physical server on an internal network, such as within a hospital network, allowing for levels of isolation and avoiding transfer of data across untrusted networks which often are requirements in the analysis of sensitive data. This was exemplified in Demonstrator 2, where a complete start-to-end workflow was run on the Galaxy platform on a secure server at Uppsala University Hospital, Sweden, leading to the identification of novel disease fingerprints in the CSF metabolome of RRMS and SPMS patients. It is worth mentioning that the selected metabolites were part of the tryptophan metabolism (alanyltryptophan and indoleacetic acid) and endocannabinoids (linoleoyl ethanolamide), both of which have been previously implicated in multiple sclerosis (Amirkhani *et al.*, 2005; Baker and Pryce, 2008; Centonze *et al.*, 2007; Lim *et al.*, 2017; Lovelace *et al.*, 2016; Zamberletti *et al.*, 2012). However, since the cross-validated predictive performance (Q2Y = 0.286) is not much higher than some of the models generated after random permutation of the response (Fig. 4A), the quality of the model needs to be confirmed in a future study on an independent cohort of larger size.

The microservice architecture is domain-agnostic and not limited to a particular assay technology, i.e. mass spectrometry. This was showcased in Demonstrator 3 and 4, where an automated 1 D NMR workflow and calculation of flux distributions (derived from the application of stable isotope resolved metabolomics) were performed. In Demonstrator 3, we showed that the pattern of the metabolite expression is different between type 2 diabetic and healthy controls, and that a large number of metabolites contribute to such separation. In Demonstrator 4, we showed a high rate of glycolysis in cells cultured in hypoxia, which is consistent with the one expected for endothelial cells (Iyer *et al.*, 1998) and with how these cells maintain energy in low oxygen environments and without oxidative phosphorylation (Eelen *et al.*, 2015; Polet and Feron, 2013). These two examples further show that complex workflows can be applied with minimal effort on other studies (i.e. simply by providing a MetaboLights accession number), leading to the capability to re-analyze data and compare the results with the original publication findings. Furthermore, it demonstrates the value of standardised dataset descriptions such as nmrML (Schober *et al.*, 2017) and ISA format (Rocca-Serra *et al.*, 2016; Sansone *et al.*, 2012) for representing NMR based studies, as well as the potential of the VRE to foster reproducibility. Furthermore, the data processing steps are trackable and replicable as each container/tool is versioned for a specific release and data processing steps and the corresponding parameters are taken care of by the workflow engine. In addition, the cli KubeNow is using speciffic pinned versions of all dependant software and all versions of software is stored in the user config dir created by the init-command. The specific version of KubeNow used is saved in user config directory.

While microservices are not confined to metabolomics and generally applicable to a large variety of applications, there are some important implications and limitations of the method. Firstly, tools need to be containerized in order to operate in the environment. This is however not particularly complex, and an increasing number of developers provide containerized versions of their tools on public container repositories such as Dockerhub or Biocontainers (da Veiga Leprevost *et al.*, 2017). Secondly, uploading data to a cloud-based system can take a considerable amount of time, and having to re-do this every time a VRE is instantiated can be time-consuming. This can be alleviated by using persistent storage on a cloud resource, but the availability of such storage varies between different cloud providers. Further, the storage system can become a bottleneck when many services try to access a shared storage. We observe that using a distributed storage system with multiple storage nodes can drastically increase performance, and the PhenoMeNal VRE comes with a distributed storage system by default. When using a workflow system to orchestrate the microservices, stability and scalability are inherently dependent on the workflow system’s job runner. Workflow execution is dependent on the underlying workflow engine, and we observed that a large number of outputs can make the Galaxy engine unresponsive, whereas the Luigi engine did not have these shortcomings. With clouds and microservices maturing, workflow systems will need to evolve and further embrace the new possibilities of these infrastructures. It is important to note that microservices do not overcome the incompatibility between tools with respect to using different data formats, and code resolving such incompatibility is still needed. However, using a shared platform makes such bridging components easier to maintain and makes them reusable. There remains great challenges in establishing interoperable and agreed-upon standards and data formats that are widely accepted and implemented by tools, as well as achieving complete support for the FAIR principles (Wilkinson *et al.*, 2016). Further, not all research can be easily pipelined, for example exploratory research might be better carried out in an ad-hoc manner than with workflows and the overhead this implies. A Jupyter Notebook as used in in Demonstrator 1 or embedded in Galaxy (Grüning *et al.*, 2017) constitutes a promising way to make use of microservices for interactive analysis. The serverless architecture, also called Functions as a Service (FaaS) architecture, is an interesting methodology when deployed with microservices as it allow developers to execute code in response to events without managing the underlying infrastructure. While serverless technologies have irrupted strongly in areas of software engineering closer to web development, this doesn’t mean that their usage can be easily transferred to scientific workloads. This is due to the far more complex network of dependencies that scientific software will have compared to web applications, where large applications can be managed for instance through npm package resolutions only. On scientific software solutions one will commonly find dependencies in different programming languages, different underlying libraries and even sometimes on different incompatible versions of the same frameworks. This level of complexity is not resolvable today through server less approaches and requires more isolated approaches based on containers, such as the one presented here.

In summary, we showed that microservices allow for efficient horizontal scaling of analyses on multiple computational nodes, enabling the processing of large datasets. By applying a number of data [mzML (Martens *et al.*, 2011), nmrML] and metadata standards [ISA serializations for study descriptions (Rocca-Serra *et al.*, 2016; Sansone *et al.*, 2012)], we also demonstrated a high level of interoperability in the context of metabolomics, by providing completely automated start-to-end analysis workflows for mass spectrometry and NMR data. In addition, many of the state-of-the-art tools such as components of XCMS ONLINE (Warth *et al.*, 2017) and MetaboAnalyst (Xia *et al.*, 2012) can be incorporated in the workflows, providing more refined workflows. The ability to instantiate VREs close to large datasets, such as on local servers within a hospital for Demonstrator 2, makes it possible to use the VRE on sensitive data that is not allowed to leave the current environment for ELSI reasons. While the current PhenoMeNal VRE implementation uses Docker for software containers and Kubernetes for container orchestration, the microservice methodology is general and not restricted to these frameworks. Likewise, the choice of Luigi and Galaxy was here used to demonstrate the capabilities of workflow management microservices in cloud environments. In fact, our microservice architecture supports other major workflow engines such as Nextflow (Di Tommaso *et al.*, 2017) or Snakemake (Köster and Rahmann, 2012). Hence it is possible to use any of such workflow engines in our VRE and still produce reproducible results. In addition, despite some of our workflows were novel in the context of metabolomics (e.g. Demonstrator 2) and can be readily applied on other datasets, their main contribution in this work is to showcase scalability and interoperability of the microservices methodology. Finally, we emphasise that the presented methodology goes beyond metabolomics and can be applied to virtually any field, lowering the barriers for taking advantage of cloud infrastructures and opening up for large-scale integrative science.

## Author contributions

KK, MAC, MC, PEK, SH contributed to Demonstrator 1. CR, KK, KP, PEK, SH, SN contributed to Demonstrator 2. KK designed the study in Demonstrator 2. JB performed collection of samples and characterization of the multiple sclerosis cohort. SH performed the mass spectrometry experiment in Demonstrator 2. DS, KP, PEK, PM, RMS, contributed to Demonstrator 3. AGB, CF, DJ, MCA, MVV, PDA, PM, PRS, SAS, TH and VS contributed to Demonstrator 4. GZ, LP, PEK and PM contributed to developments of Galaxy in Kubernetes. AL and MC contributed to the development of Luigi in Kubernetes. AL, MAC, MC and NS developed KubeNow. PM contributed to Galaxy-Kubernetes. EAT and PR contributed to containerizing of Workflow4Metabolomics tools. AGB, DJ, PRS and SAS contributed to ISA-API. DJ, EAT, KP, MVV, NS, OS, PEK, PM, PR, PRS, DS, RMS, RR and SB were involved in testing the containers and the VRE. PM, SIH and KH were involved in development and maintenance of the portal. MVV, PM and RMS contributed to the release. NK coordinated the PhenoMeNal project. CS conceived and managed the PhenoMeNal project. OS conceived and coordinated the study and e-infrastructure. All authors contributed to manuscript writing.

## Funding

This research was supported by The European Commission’s Horizon 2020 programme funded under grant agreement number 654241 (PhenoMeNal), The Swedish Research Council FORMAS, Uppsala Berzelii Technology Centre for Neurodiagnostics, Åke Wiberg Foundation and the Nordic e-Infrastructure Collaboration (NeIC) via the Glenna2 and Tryggve2 projects. We kindly acknowledge contributions by Daniel Jacob (INRA) and to cloud resources by SNIC Science Cloud, Embassy Cloud, c-Pouta and CityCloud. The funders had no role in study design, data collection and analysis, decision to publish, or preparation of the manuscript.

*Conflict of Interest*: none declared.

## Supplementary Material

btz160_Supplementary_Materials
