# Supplementary material for: Interoperable and scalable data analysis with microservices: applications in metabolomics
Source: Bioinformatics. 2019 Mar 9;35(19):3752–60. doi: 10.1093/bioinformatics/btz160 (PMC6761976; doi:10.1093/bioinformatics/btz160)
Supplement: btz160_Supplementary_Materials [file btz160_supplementary_materials.zip › btz160-suppl_data/Supplementary_Methods.pdf]

Supplementary methods

Containers

Our microservice-based architecture uses Docker (https://www.docker.com/) containers to encapsulate software tools. PhenoMeNal produces guidelines on the release process to be followed by developers for each container (see the PhenoMeNal wiki; https://github.com/phnmnl/phenomenal-h2020/wiki). For a container image built to be pushed to the PhenoMeNal container registry (container-registry.phenomenal-h2020.eu/; see Figure S1), it must first pass simple testing criteria regarding the correct provision and executability of defined binaries inside the container. Containers tagged for release are also pushed to the BioContainers Docker Hub (https://hub.docker.com/r/biocontainers/). All published containers are thus available for download and can be used in any microservice architecture.

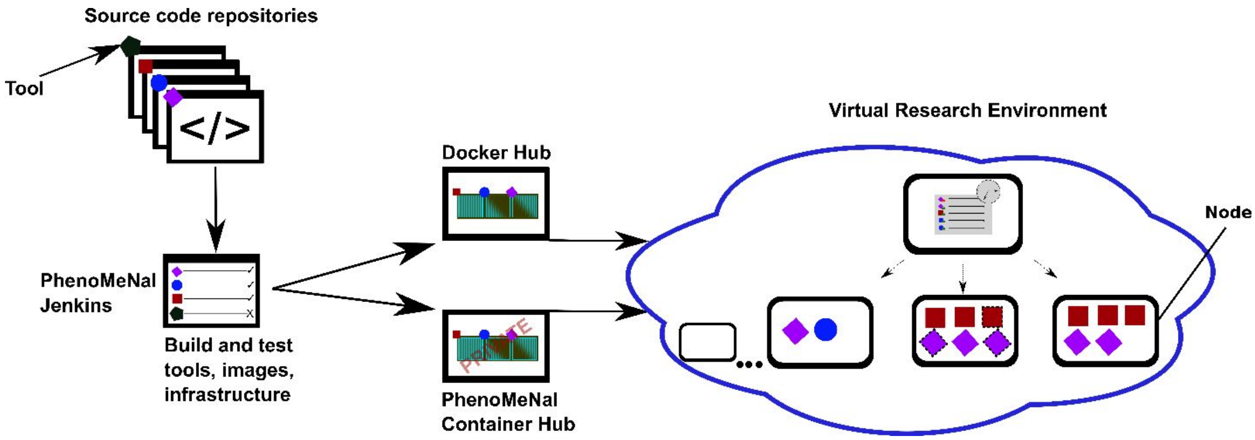

Figure S1: Overview of the continuous development and operation in PhenoMeNal. Source code for tools are pulled into a continuous integration system (Jenkins), where the containers are assembled, tested, and if passing the tests pushed to one or more container repositories from where they are made available to the VREs. While the depiction of the PhenoMeNal registry says private, this only means that it is private for depositing containers; the containers on the PhenoMeNal container registry are publicly available for anyone to use them.

PhenoMeNal Virtual Research Environment (VRE)

The PhenoMeNal VRE is a virtual environment based on the Kubernetes container orchestrator (https://kubernetes.io/). Container orchestration includes initialisation and scaling of jobs based on containers, abstractions to file system access for running containers, exposure of services within and outside the VRE, safe provisioning of secrets to running containers, resource usage administration, scheduling of new container based jobs as well as rescheduling of failed jobs and long running services (all based on containers) to ensure that a desired state is maintained (ie. n number of Galaxy instances must be running).

There are two main classes of services in a PhenoMeNal VRE: long-lasting services, and compute jobs. Long-lasting services are applications that need to be continuously up and running (e.g. a user interface, like the Galaxy workflow environment), while compute jobs are short-lived services that perform temporary functions in data processing and thus are only executed on demand and destroyed afterwards. Long-lasting services and jobs are implemented using both Kubernetes deployments and replication controllers, while jobs are implemented using Kubernetes jobs. The deployment of long term running services is normally more complex in terms of number of components (deployment/replication controller, service, secrets, configuration maps, front end container, database container, etc) compared to compute jobs (which rely only on a single container, a command and access to read data and write results). As such, PhenoMeNal uses the Helm package manager (https://github.com/kubernetes/helm) for Kubernetes, and has produced Helm charts for Galaxy, Jupyter, the PhenoMeNal Portal (towards automatic deployments behind firewalls) and Luigi. The use of Helm means that PhenoMeNal long-term services, and by extension the compute jobs triggered by them, can be executed on any Kubernetes cluster with access to a shared file system, and not only those provisioned by PhenoMeNal.

Kubernetes does not cover host cloud and virtual machine provisioning. For this reason, we developed KubeNow (https://github.com/kubenow/KubeNow), which is a solution based on Terraform (https://www.terraform.io/), Ansible (https://www.ansible.com/) and kubeadm (https://github.com/kubernetes/kubeadm). KubeNow makes it easy to instantiate a complete virtual

infrastructure of compute nodes, shared file system storage, networks, configure DNS, operating system, container implementation and orchestration tools on a local computer or server (via VirtualBox or KVM), a private cloud (e.g. OpenStack), or a public cloud provider (e.g. Google Cloud, Amazon Web Services, Microsoft Azure).

A key objective of the VRE is to relieve the user from the burden of setting up the necessary IT-infrastructure of hardware and software for analysis. The VRE allows users to take advantage of the microservices inside the VRE in several ways: a) directly starting a single application inside a container, b) wrapping containers in an analysis script, or the promoted way c) integrating it into a new or existing scientific workflow (a chain of containerized tools that serially process input data). In order to launch a PhenoMeNal VRE, users can either take advantage of a PhenoMeNal client for KubeNow (https://github.com/phnmnl/cloud-deploy-kubenow) or use the PhenoMeNal web portal (https://portal.phenomenal-h2020.eu) that allows users to launch VREs on the largest public cloud providers.

The main workflow interface and engine is Galaxy (https://galaxyproject.org/), as shown in Demonstrators 2, 3 and 4, but users can also use Luigi (https://github.com/spotify/luigi) and Jupyter (http://jupyter.org/) (if more interactive scripting/scientific computing is required) as presented in Demonstrator 1. Both workflow systems have been adapted to run jobs on top of Kubernetes, and these contributions are now integrated and maintained in the core Galaxy and Luigi projects. Apart from the VRE, PhenoMeNal makes available a suite of well-tested and interoperable containers for metabolomics (see Supplementary Figure S1).

In addition to GUIs, workflows can be accessed, executed and tested via APIs with wft4galaxy (https://github.com/phnmnl/wft4galaxy). Here, the user can send data and queries to Galaxy from the command line either manually or with macro-files and test the workflow and expected output for reproducibility. In order to access the API, an API key for the desired account must be generated from within Galaxy. Requiring the API key ensures only authorized access to the API. In this way, demonstrators 2, 3 and 4 can be invoked from the command line, can be tested for reproducible output and executed with different parameters and data.

Demonstrator 1:

The original analysis workflow by C. Ranninger et al. [1], whose HPLC-MS data was deposited in ISA-Tab format (Rocca-Serra *et al.*, 2010) to MetaboLights (MetaboLights ID: MTBLS233), was containerized using Docker. In the study, the authors reported the effect of scan parameters in the number of detected features in an Orbitrap-based mass spectrometer. More specifically, the authors argued and proved that having independent parameters in different mass ranges can greatly improve the ability of MS to detect metabolites. The experiment was based on 27 samples from lysates of a human kidney cell line, six pooled samples and eleven blank samples (total of 44 unique samples). Each of the 44 samples was run using mass spectrometry with three different settings for both positive and negative ionization:

1) a **full scan** by which the analytes were quantified in a single m/z range 50–1000 (for each sample one MS data file was produced).

2) **alternating scan** approach in which the mass range was split into m/z 50–200 and m/z 200–1000 utilizing the same parameters as the first approach (for each sample three MS data files were produced).

3) in this **separate** approach, each sample was run two times, one run for low (m/z 50–200) and one for high range masses (m/z 200–1000) using two different settings (for each sample two MS data files were produced).

In addition to these three approaches, an optimized and conclusional run was made, generating one file per sample, based on the result from the three tested approaches. Considering both positive and negative ionization, 12 files were produced for each of the 44 samples, resulting in a total of 528 MS files. In other words, these 528 MS files can be placed into 12 groups, each corresponding to one of approaches mentioned above (note that the alternating scan produced 3 and the separate approach produced 2 groups). Each of these groups included 44 samples.

Original preprocessing workflow

The preprocessing workflow was based on the OpenMS platform (Sturm *et al.*, 2008) by which the raw data files were first centroided using PeakPickerHiRes, the mass traces were quantified using

FeatureFinderMetabo and were matched across the data files using FeatureLinkerUnlabeledQT. The parameters for these tools were individually set for positive and negative ionization. In addition, FileFilter was used to filter out certain features having retention time lower than 39 seconds and being present in at least six samples and finally the list of features was exported to text format using TextExporter. The downstream analysis was performed using R (R Development Core Team, 2008) on the KNIME workflow engine (Berthold *et al.*, 2009).

Preprocessing workflow using microservices and Luigi

OpenMS version 1.11.1 was containerized using the build instructions provided in the OpenMS Github repository for Linux (https://github.com/OpenMS/OpenMS/wiki/Building-OpenMS). Briefly, the OpenMS dependencies were first installed followed by building "contrib" libraries as well as OpenMS core. The docker file is publicly available in "https://github.com/phnmnl/container-openms/blob/mtbls233/Dockerfile" and can be pulled and run from "docker-registry.phenomenal-h2020.eu/phnmnl/openms". The file OpenMS tasks that were using Luigi, ran in parallel over the 12 groups, each consisting of 44 MS files corresponding to one specific setting.

The first two tasks, peak picking and feature finding, were perfectly parallelizable, hence we executed them concurrently for all the samples using 2 gigabytes of RAM and 1 CPU. However, feature linking matches the features across corresponding runs, meaning that the samples with identical MS settings needed to be linked together and separate from the samples with different settings. Therefore, we performed 12 of these tasks in parallel. In order to maximize parallelization, each feature linker was run using 4 gigabytes of RAM and 2 CPUs. The linking tool produced a single file for each the 12 groups which were then processed independently by the last two tasks: file filter and text exporter both of which were performed using 2 gigabytes of RAM and 1 CPU. All compute nodes were connected to a shared clustered Gluster filesystem (https://www.gluster.org).

Weak Scaling Efficiency (WSE)

The Weak Scaling Efficiency (WSE) is a measure of how good a concurrent program scales computationally, when the same workload (or problem size) is assigned to each processor and additional processors are added to solve a larger problem. Given the full MTBLS233 dataset we ran the

preprocessing analysis on 40 Luigi workers. Then for 1/4, 2/4, 3/4 of MTBLS233 we ran again on 10, 20 and 30 workers respectively. For each run we measured the processing time T_{10}, T_{20}, T_{30} and T_{40}, and we computed the WSE as:

$$WSE_n = \frac{T_{10}}{T_n} \; for \; n = 10, \; 20, \; 30, \; 40$$

The downstream analysis, previously implemented in R as KNIME nodes, were extracted and moved to the interactive and responsive environment of Jupyter notebook using the provided R kernel. The deployment and preprocessing as well as the downstream analysis can be accessed on https://github.com/phnmnl/MTBLS233-Jupyter

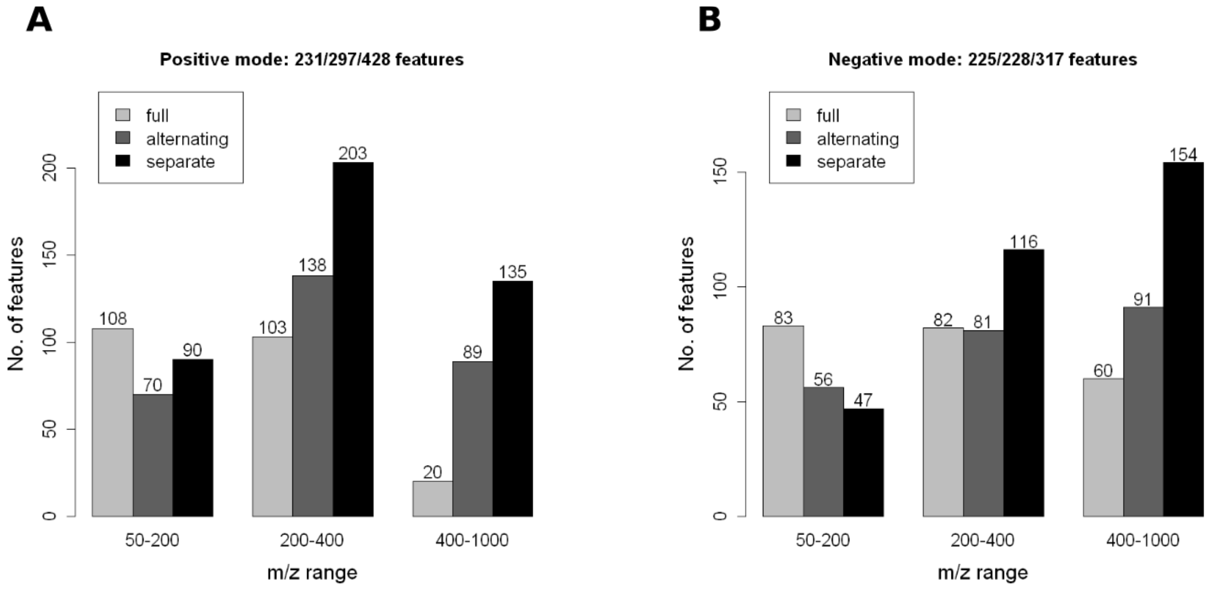

Figure S2. Result of downstream analysis on MTBLS233 dataset. A) Number of features quantified in positive ionization mode. B) Number of features quantified in negative ionization mode.

Demonstrator 2:

Mass spectrometry data processing is complex and normally requires a combination of many different tools e.g. to reduce the noise, perform quantification, alignment, matching, identification and statistics. This procedure becomes even more sophisticated considering that there is a large share of different algorithms and tools for each of the mentioned steps. This indeed requires substantial technical knowledge of each tool including installation, format of input/output, and the parameters. The purpose of this demonstrator was to show the capability of the microservice architecture to eliminate the need for installation and converting data formats. Although, this demonstrator does not concern parameter optimization, a standard, well tested workflow can be reused for many type of instruments and experiments with minimal changes needed, given it has a standardized exchange data format.

The data used in this demonstrator includes 37 clinical CSF samples of thirteen relapsing-remitting multiple sclerosis (RRMS) and fourteen secondary progressive multiple sclerosis (SPMS) patients as well as ten controls (non-multiple sclerosis). 26 quality controls including 19 blank and seven dilution series samples were also added to the experiment. In addition, 8 MS/MS samples were included in the experiment for identification. The samples were processed and analyzed using the LC-MS workflow illustrated in Figure S3.

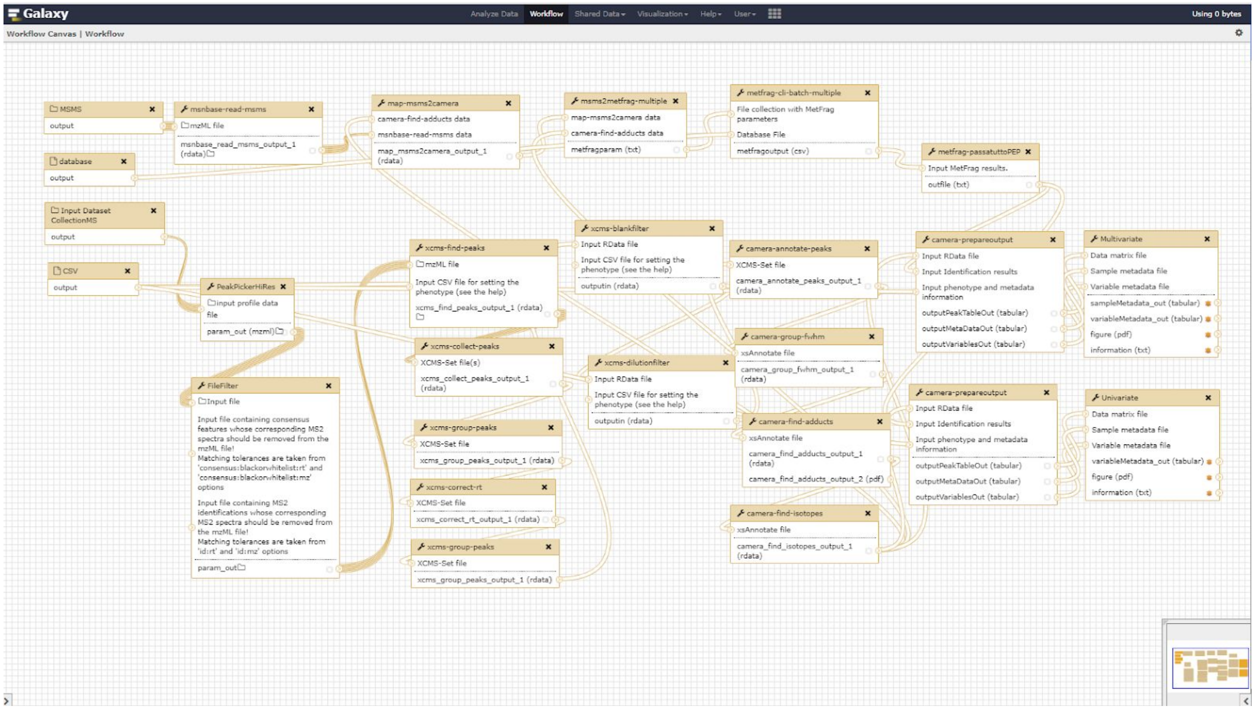

Figure S3. Overview of the mass spectrometry workflow in Galaxy used for demonstrator 2.

The LC-MS samples in vendor specific format (RAW) were first converted to the open access standard mzML using “msconvert” and then processed through the following workflow in Galaxy to perform the quantification:

Noise reduction and filtering (OpenMS)

The data was centroided using “PeakPickerHiRes” (default parameters) and intensity maps were cropped using “FileFilter” implemented in OpenMS Docker container (https://github.com/phnmnl/container-openms). All the OpenMS nodes in Galaxy were generated using CTDConverter (https://github.com/WorkflowConversion/CTDConverter).

Quantification, alignment and matching (XCMS)

The quantification was performed using XCMS container (https://github.com/phnmnl/container-xcms) such that the mass traces were identified and quantified with “findPeaks”. In addition to the filtered mzML files, this node accepts a CSV (comma-separated value) file by which users can add phenotypic (class)

information for each of the samples. Afterwards, the retention time drifts between samples were adjusted using "retcor" and the mass traces were grouped across the samples using "group" function in XCMS.

Further filtering of biological non-relevant signals (R)

Using the same approach as "PeakML.BlankFilter" and "PeakML.DilutionTrendFilter" from mzMatch(Scheltema *et al.*, 2011), two containers were created to filter out biologically non-relevant and spurious signals based on the blank and dilution series samples. More specifically, "blankfilter" accepts the output of grouping (XCMS) and based on the user defined parameters, signals with higher abundance in the non-biological samples (blanks) will be removed from the dataset. Dilution series filtering (dilutionfilter) on the other hand, removes signals if they do not correlate with a user defined series (i.e. a series in which the amount of sample analyzed was sequentially increased). Both of these nodes can accept a table showing which files the algorithm must use as blank and biological samples (https://github.com/phnmnl/container-xcms).

Annotation of signals (CAMERA)

Annotation of the peaks was performed using CAMERA container (https://github.com/phnmnl/container-camera) by applying the following functions: "groupFWHM", "findAdducts" and "findIsotopes". This part of the workflow attempts to group the metabolites based on their isotopes as well as adduct information.

Identification (MetFrag)

To perform the metabolite identification, the tandem spectra from the MS2 samples in mzML format were extracted using MSnbase (https://github.com/phnmnl/container-MSnbase) container ("metfrag-read-msms") and parent ion m/z were converted to neutral mass based on the adducts and isotope information from CAMERA ("map-msms2camera" and "msms2metfrag"). The resulting neutral mass and the tandem information were used in MetFrag (https://github.com/phnmnl/container-metfrag-cli-batch) container ("metfrag-cli-multiple") to perform *in silico* fragmentation and scoring of the metabolite candidates retrieved from the human metabolome database (HMDB version 2017-07) (Wishart *et al.*, 2007). The MetFrag scores were converted to q-values using Passatutto (https://github.com/phnmnl/container-passatutto) software ("metfrag-passatutoPEP").

Various parameters can be set in this part of the workflow including type of adducts, desired m/z and RT deviation as well as several options regarding MetFrag identification and score transformation.

Statistics (Workflow4Metabolomics)

The result of identification and quantification were used in “camera-prepareoutput”. This node accepts the quantification (CAMERA results), identification (MetFrag results) and sample metadata (CSV file) and prepares the output for “Multivariate” and “Univariate” containers (https://github.com/phnmnl/container-multivariate and https://github.com/phnmnl/container-univariate) from Workflow4Metabolomics to perform a PLS-DA. In addition, analysis of variance (ANOVA) was performed to select the metabolites with altered levels. We used specific cutoff of 0.05 both on f-statistics and t-statistics (comparing RRMS and SPMS to controls) to select the metabolites. Finally, only the metabolites were limited to those that were identified and quantified in human based on HMDB information.

Demonstrator 3:

The purpose of this workflow was to demonstrate application of 1D NMR data analysis using microservices. The workflow illustrated in Figure S4 was implemented in Galaxy and applied to the MTBLS1 data set imported from the MetaboLights database.

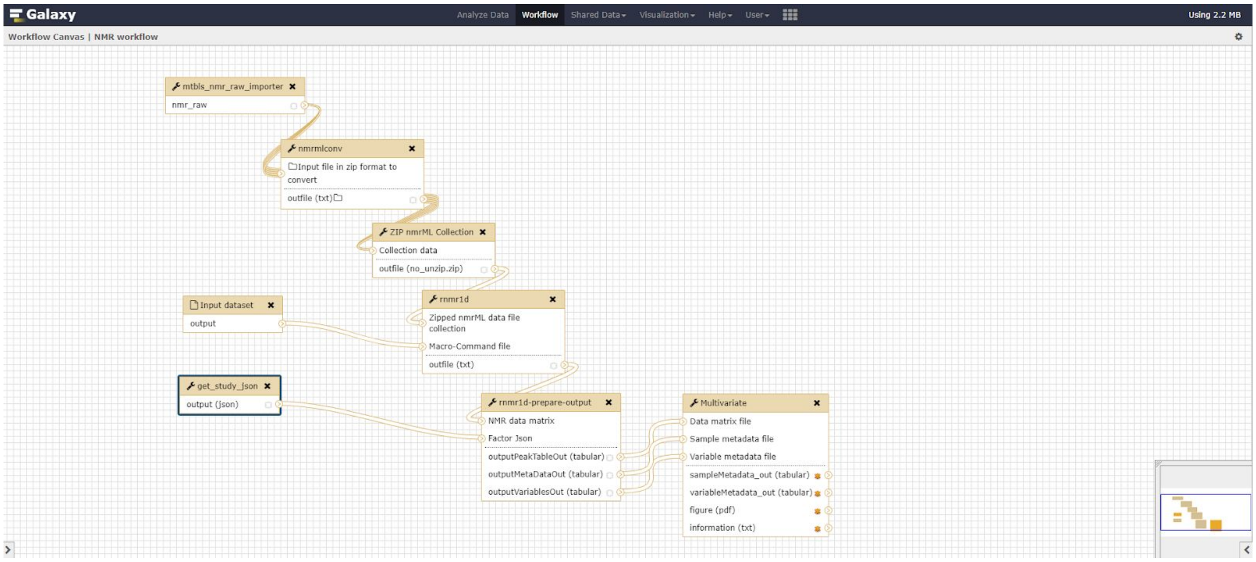

Figure S4. 1D NMR data analysis workflow, exemplifying secondary data usage through import from a dedicated metabolomics repository.

The NMR vendor data files are first downloaded using “mtbls_nmr_raw_import” and converted to nmrML format using “nmrmlconv”. The resulting open access data are then zipped (“ZIP nmrML Collection”) and fed into “rnmr1d” (https://github.com/phnmnl/container-rnmr1d). This specific node requires a macro settings file (which can be obtained from https://github.com/PayamEmami/galaxyWorkflows). Afterwards, the container performs the fourier transformation (ftt), zero-filling, line broadening, phase-correction and baseline correction, resulting to a peak matrix. At the same time “get_study_json” must be used to extract all metadata information from a specified MetaboLights study to a JSON representation of the ISA formatted metadata (https://github.com/phnmnl/container-mtblisa). The peak matrix and the JSON file are used in “rnmr1d-prepare-output” which prepares the output consisting of all available metadata information for “Multivariate” or “Univariate” nodes for performing statistics on factor of interest.

Demonstrator 4:

In this GC-MS-based metabolomics study, HUVEC (Human Umbilical Vein Endothelial Cell) cells were incubated for 40 hours in the presence of [1,2-$^{13}C_2$]glucose and ^{13}C propagation to glycogen, RNA ribose and lactate was used to constrain the flux values through glycolysis and pentose -phosphate pathways.

The workflow illustrated in Figure S6 was implemented in Galaxy. The fluxomics data analysis workflow corresponding to Figure 6 is available in the PhenoMeNal VRE as a shared workflow. HUVEC cell data are available on the MTBLS412 data set that can be imported from the MetaboLights database. ^{13}C Metabolic Flux Analysis (13C MFA), for estimation of metabolic reaction-fluxes through the integration of ^{13}C metabolomics data, is achieved by using Iso2Flux to maximize the consistency between flux-balanced model predictions and experimental mass spectrometry measurements. The flux values (numerical values associated with the metabolic scheme in Figure 6) refers to the output of lactate, as lactate was the only fixed value set to 1. The generated flux map showed that most of the secreted lactate derived from the consumed glucose, with a comparatively low pass through pentose-phosphate pathway.

The corresponding tools are:

- RaMID (https://github.com/seliv55/RaMID): An R-based tool for reading and extracting the necessary information from mass spectrometer generated netCDF files. It extracts mass spectra of the metabolites of interest corresponding to the m/z and retention time indicated in an input file, corrects baseline, and saves the resulting data.

- MIDcor (https://github.com/seliv55/midcor) an R-based tool that takes the output of RaMID and corrects the evaluated mass spectra for natural isotopes abundance and possible peak overlapping as described in(Selivanov *et al.*, 2017) and computes isotopologue fractions.

- Iso2Flux (https://github.com/cfoguet/iso2flux): a python-based ^{13}C MFA tool. Iso2Flux uses constraint-based modelling and the EMU Elementary Metabolite Unit framework (Antoniewicz *et al.*, 2007) to compute steady-state distribution of ^{13}C label as function of steady-state flux distribution. Fitting to experimental data provides the distribution of underlying metabolic fluxes. Iso2Flux, takes the output of MIDcor, an SBML file defining the metabolic network and a CSV file defining the ^{13}C propagation rules and generates the following outputs: a) the simulated fluxes that are most consistent with experimental

^{13}C data, b) the simulated label obtained with such fluxes, c) lower and upper limits of the 95% confidence intervals for fluxes e) an SBML model constrained with the results of the ^{13}C analysis.

- Escher Fluxomics (https://github.com/escher/escher-demo): a web-based tool for visualisation of reaction-fluxes in relation to pathways.

Containers have been compiled for these tools and galaxy wrappers have been developed for them. These tools, together with the ProteoWizard container, have been used for the creation of the workflow in Galaxy (figure S5).

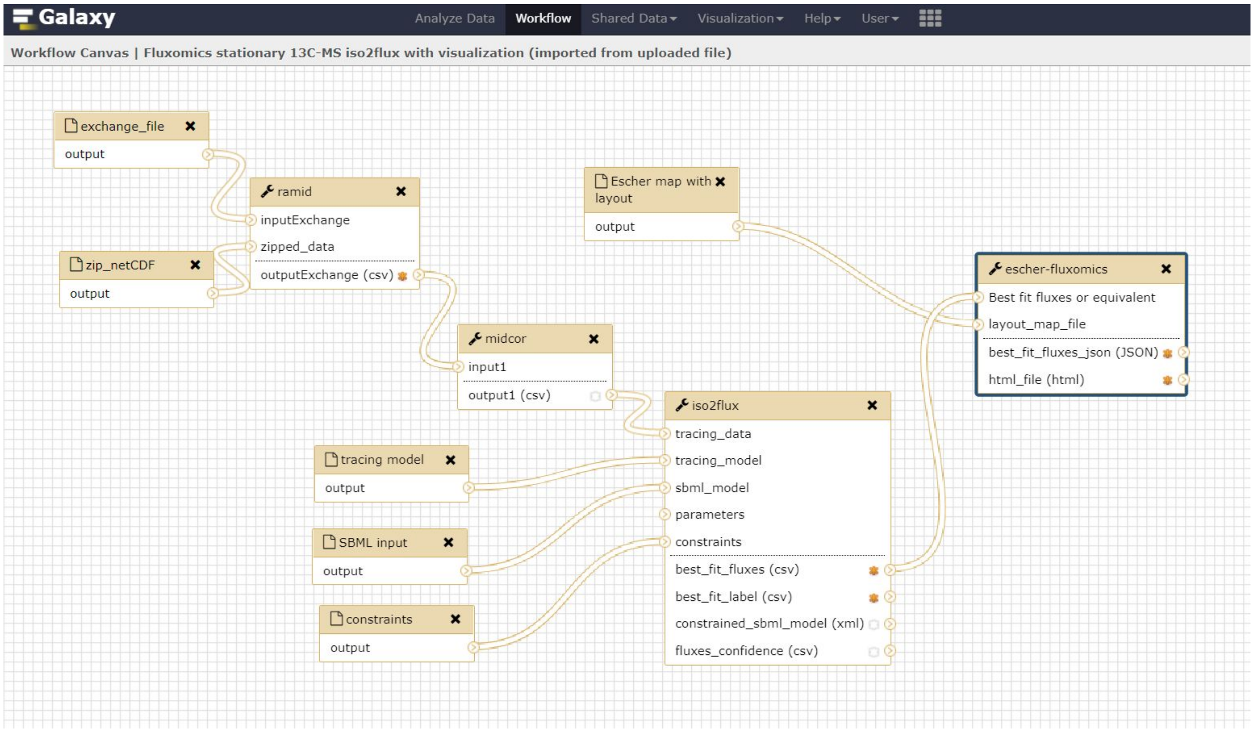

Figure S5: Fluxomics data analysis workflow from ^{13}C tracer-based data written by a mass spectrometer in netCDF files. The workflow connects the tools RaMID, MIDcor, Iso2flux and Escher Fluxomics.

References

Antoniewicz,M.R. *et al.* (2007) Elementary metabolite units (EMU): a novel framework for modeling isotopic distributions. *Metab. Eng.*, **9**, 68–86.

Berthold,M.R. *et al.* (2009) KNIME - the Konstanz information miner. *ACM SIGKDD Explorations Newsletter*, **11**, 26.

R Development Core Team (2008) R: A Language and Environment for Statistical Computing.

Rocca-Serra,P. *et al.* (2010) ISA software suite: supporting standards-compliant experimental annotation and enabling curation at the community level. *Bioinformatics*, **26**, 2354–2356.
Scheltema,R.A. *et al.* (2011) PeakML/mzMatch: a file format, Java library, R library, and tool-chain for mass spectrometry data analysis. *Anal. Chem.*, **83**, 2786–2793.
Selivanov,V.A. *et al.* (2017) MIDcor, an R-program for deciphering mass interferences in mass spectra of metabolites enriched in stable isotopes. *BMC Bioinformatics*, **18**, 88.
Sturm,M. *et al.* (2008) OpenMS - an open-source software framework for mass spectrometry. *BMC Bioinformatics*, **9**, 163.
Wishart,D.S. *et al.* (2007) HMDB: the Human Metabolome Database. *Nucleic Acids Res.*, **35**, D521–D526.
