# Supplementary material for: Interoperable and scalable data analysis with microservices: applications in metabolomics
Source: Bioinformatics. 2019 Mar 9;35(19):3752–60. doi: 10.1093/bioinformatics/btz160 (PMC6761976; doi:10.1093/bioinformatics/btz160)
Supplement: btz160_Supplementary_Materials [file btz160_supplementary_materials.zip › btz160-suppl_data/TableS1.pdf]

Tool ID	Tool.Container	Category	GitHub	Reference
escher-fluxomics	container-escher-fluxomics	Fluxomics Tools	https://github.com/phnmnl/container-escher-fluxomics	Not Available
iso2flux	container-iso2flux	Fluxomics Tools	https://github.com/phnmnl/container-iso2flux	Not Available
midcor	container-midcor	Fluxomics Tools	https://github.com/phnmnl/container-midcor	(Selivanov *et al.*, 2017)
ramid	container-ramid	Fluxomics Tools	https://github.com/phnmnl/container-ramid	Not Available
cdf2mid	container-cdf2mid	Fluxomics Tools	https://github.com/phnmnl/container-cdf2mid	Not Available
isa_factors_viz	container-mtbls-factors-viz	Data	https://github.com/phnmnl/container-mtbls-factors-viz	(Sansone *et al.*, 2016, 2012)
power-analysis	container-papy	Statistical Analysis	https://github.com/phnmnl/container-papy	(Blaise *et al.*, 2016)
mtbls-dwnld	container-mtbls-dwnld	Data	https://github.com/phnmnl/container-mtbls-dwnld	(González-Beltrán *et al.*, 2014; Giacomoni *et al.*, 2015)
isa2mzml	container-isa-extractor	Data	https://github.com/phnmnl/container-isa-extractor	(González-Beltrán *et al.*, 2014; Giacomoni *et al.*, 2015)
isa2mzxml	container-isa-extractor	Data	https://github.com/phnmnl/container-isa-extractor	(González-Beltrán *et al.*, 2014; Giacomoni *et al.*, 2015)
isa2mzdata	container-isa-extractor	Data	https://github.com/phnmnl/container-isa-extractor	(González-Beltrán *et al.*, 2014; Giacomoni *et al.*, 2015)
isa2nmrml	container-isa-extractor	Data	https://github.com/phnmnl/container-isa-extractor	(González-Beltrán *et al.*, 2014; Giacomoni *et al.*, 2015)
isa2netcdf	container-isa-extractor	Data	https://github.com/phnmnl/container-isa-extractor	(González-Beltrán *et al.*, 2014; Giacomoni *et al.*, 2015)
isa2w4m	container-isa2w4m	Data	https://github.com/phnmnl/container-isa2w4m	(Sansone *et al.*, 2012; Giacomoni *et al.*, 2015)

normalization	container-normalization	Statistical Analysis Tools	https://github.com/phnmnl/container-normalization	(Dieterle *et al.*, 2006)
Transformation	container-transformation	Statistical Analysis Tools	https://github.com/phnmnl/container-transformation	(Veselkov *et al.*, 2011)
Batch_correction	container-batch_correction	Statistical Analysis Tools	https://github.com/phnmnl/container-batch_correction	(van der Kloet *et al.*, 2009; Dunn *et al.*, 2011; Thevenot *et al.*, 2015)
generic_filter	container-tool-generic_filter	Statistical Analysis Tools	https://github.com/phnmnl/container-tool-generic_filter	(Giacomoni *et al.*, 2015)
quality_metrics	container-qualitymetrics	Statistical Analysis Tools	https://github.com/phnmnl/container-qualitymetrics	(Giacomoni *et al.*, 2015; Mason *et al.*, 1997; Thevenot *et al.*, 2015)
biosigner	container-biosigner	Statistical Analysis Tools	https://github.com/phnmnl/container-biosigner	(Rinaudo *et al.*, 2016)
AdditiveSeries	container-openms	Mass Spectrometry	https://github.com/phnmnl/container-openms	(Sturm *et al.*, 2008)
BaselineFilter	container-openms	Mass Spectrometry	https://github.com/phnmnl/container-openms	(Sturm *et al.*, 2008)
CompNovo	container-openms	Mass Spectrometry	https://github.com/phnmnl/container-openms	(Sturm *et al.*, 2008)
CompNovoCID	container-openms	Mass Spectrometry	https://github.com/phnmnl/container-openms	(Sturm *et al.*, 2008)
ConsensusID	container-openms	Mass Spectrometry	https://github.com/phnmnl/container-openms	(Sturm *et al.*, 2008)
ConsensusMapNormalizer	container-openms	Mass Spectrometry	https://github.com/phnmnl/container-openms	(Sturm *et al.*, 2008)
Decharger	container-openms	Mass Spectrometry	https://github.com/phnmnl/container-openms	(Sturm *et al.*, 2008)
DTAExtractor	container-openms	Mass Spectrometry	https://github.com/phnmnl/container-openms	(Sturm *et al.*, 2008)
EICExtractor	container-openms	Mass Spectrometry	https://github.com/phnmnl/container-openms	(Sturm *et al.*, 2008)

ExternalCalibration	container-openms	Mass Spectrometry	https://github.com/phnmnl/container-openms	(Sturm *et al.*, 2008)
FalseDiscoveryRate	container-openms	Mass Spectrometry	https://github.com/phnmnl/container-openms	(Sturm *et al.*, 2008)
FeatureFinderCentroided	container-openms	Mass Spectrometry	https://github.com/phnmnl/container-openms	(Sturm *et al.*, 2008)
FeatureFinderIdentification	container-openms	Mass Spectrometry	https://github.com/phnmnl/container-openms	(Sturm *et al.*, 2008)
FeatureFinderIsotopeWavelet	container-openms	Mass Spectrometry	https://github.com/phnmnl/container-openms	(Sturm *et al.*, 2008)
FeatureFinderMetabo	container-openms	Mass Spectrometry	https://github.com/phnmnl/container-openms	(Sturm *et al.*, 2008)
FeatureFinderMRM	container-openms	Mass Spectrometry	https://github.com/phnmnl/container-openms	(Sturm *et al.*, 2008)
FeatureFinderMultiplex	container-openms	Mass Spectrometry	https://github.com/phnmnl/container-openms	(Sturm *et al.*, 2008)
FeatureLinkerLabeled	container-openms	Mass Spectrometry	https://github.com/phnmnl/container-openms	(Sturm *et al.*, 2008)
FeatureLinkerUnlabeled	container-openms	Mass Spectrometry	https://github.com/phnmnl/container-openms	(Sturm *et al.*, 2008)
FeatureLinkerUnlabeledQT	container-openms	Mass Spectrometry	https://github.com/phnmnl/container-openms	(Sturm *et al.*, 2008)
FidoAdapter	container-openms	Mass Spectrometry	https://github.com/phnmnl/container-openms	(Sturm *et al.*, 2008)
FileConverter	container-openms	Mass Spectrometry	https://github.com/phnmnl/container-openms	(Sturm *et al.*, 2008)
FileFilter	container-openms	Mass Spectrometry	https://github.com/phnmnl/container-openms	(Sturm *et al.*, 2008)
FileInfo	container-openms	Mass Spectrometry	https://github.com/phnmnl/container-openms	(Sturm *et al.*, 2008)
FileMerger	container-openms	Mass Spectrometry	https://github.com/phnmnl/container-openms	(Sturm *et al.*, 2008)
HighResPrecursorMassCorrector	container-openms	Mass Spectrometry	https://github.com/phnmnl/container-openms	(Sturm *et al.*, 2008)
IDConflictResolver	container-openms	Mass Spectrometry	https://github.com/phnmnl/container-openms	(Sturm *et al.*, 2008)
IDFileConverter	container-openms	Mass Spectrometry	https://github.com/phnmnl/container-openms	(Sturm *et al.*, 2008)

IDFilter	container-openms	Mass Spectrometry	https://github.com/phnmnl/container-openms	(Sturm *et al.*, 2008)
IDMapper	container-openms	Mass Spectrometry	https://github.com/phnmnl/container-openms	(Sturm *et al.*, 2008)
IDMerger	container-openms	Mass Spectrometry	https://github.com/phnmnl/container-openms	(Sturm *et al.*, 2008)
IDPosteriorErrorProbability	container-openms	Mass Spectrometry	https://github.com/phnmnl/container-openms	(Sturm *et al.*, 2008)
IDRipper	container-openms	Mass Spectrometry	https://github.com/phnmnl/container-openms	(Sturm *et al.*, 2008)
IDRTCalibration	container-openms	Mass Spectrometry	https://github.com/phnmnl/container-openms	(Sturm *et al.*, 2008)
InclusionExclusionListCreator	container-openms	Mass Spectrometry	https://github.com/phnmnl/container-openms	(Sturm *et al.*, 2008)
InspectAdapter	container-openms	Mass Spectrometry	https://github.com/phnmnl/container-openms	(Sturm *et al.*, 2008)
InternalCalibration	container-openms	Mass Spectrometry	https://github.com/phnmnl/container-openms	(Sturm *et al.*, 2008)
IsobaricAnalyzer	container-openms	Mass Spectrometry	https://github.com/phnmnl/container-openms	(Sturm *et al.*, 2008)
ITRAQAnalyzer	container-openms	Mass Spectrometry	https://github.com/phnmnl/container-openms	(Sturm *et al.*, 2008)
LuciphorAdapter	container-openms	Mass Spectrometry	https://github.com/phnmnl/container-openms	(Sturm *et al.*, 2008)
MapAlignerIdentification	container-openms	Mass Spectrometry	https://github.com/phnmnl/container-openms	(Sturm *et al.*, 2008)
MapAlignerPoseClustering	container-openms	Mass Spectrometry	https://github.com/phnmnl/container-openms	(Sturm *et al.*, 2008)
MapAlignerSpectrum	container-openms	Mass Spectrometry	https://github.com/phnmnl/container-openms	(Sturm *et al.*, 2008)
MapNormalizer	container-openms	Mass Spectrometry	https://github.com/phnmnl/container-openms	(Sturm *et al.*, 2008)
MapRTTransformer	container-openms	Mass Spectrometry	https://github.com/phnmnl/container-openms	(Sturm *et al.*, 2008)
MapStatistics	container-openms	Mass Spectrometry	https://github.com/phnmnl/container-openms	(Sturm *et al.*, 2008)
MascotAdapter	container-openms	Mass Spectrometry	https://github.com/phnmnl/container-openms	(Sturm *et al.*, 2008)

MascotAdapterOnline	container-openms	Mass Spectrometry	https://github.com/phnmnl/container-openms	(Sturm *et al.*, 2008)
MassTraceExtractor	container-openms	Mass Spectrometry	https://github.com/phnmnl/container-openms	(Sturm *et al.*, 2008)
MRMMapper	container-openms	Mass Spectrometry	https://github.com/phnmnl/container-openms	(Sturm *et al.*, 2008)
MSGFPlusAdapter	container-openms	Mass Spectrometry	https://github.com/phnmnl/container-openms	(Sturm *et al.*, 2008)
MyriMatchAdapter	container-openms	Mass Spectrometry	https://github.com/phnmnl/container-openms	(Sturm *et al.*, 2008)
MzTabExporter	container-openms	Mass Spectrometry	https://github.com/phnmnl/container-openms	(Sturm *et al.*, 2008)
NoiseFilterGaussian	container-openms	Mass Spectrometry	https://github.com/phnmnl/container-openms	(Sturm *et al.*, 2008)
NoiseFilterSGolay	container-openms	Mass Spectrometry	https://github.com/phnmnl/container-openms	(Sturm *et al.*, 2008)
OMSSAAdapter	container-openms	Mass Spectrometry	https://github.com/phnmnl/container-openms	(Sturm *et al.*, 2008)
OpenSwathAnalyzer	container-openms	Mass Spectrometry	https://github.com/phnmnl/container-openms	(Sturm *et al.*, 2008)
OpenSwathAssayGenerator	container-openms	Mass Spectrometry	https://github.com/phnmnl/container-openms	(Sturm *et al.*, 2008)
OpenSwathChromatogramExtractor	container-openms	Mass Spectrometry	https://github.com/phnmnl/container-openms	(Sturm *et al.*, 2008)
OpenSwathConfidenceScoring	container-openms	Mass Spectrometry	https://github.com/phnmnl/container-openms	(Sturm *et al.*, 2008)
OpenSwathDecoyGenerator	container-openms	Mass Spectrometry	https://github.com/phnmnl/container-openms	(Sturm *et al.*, 2008)
OpenSwathFeatureXMLToTSV	container-openms	Mass Spectrometry	https://github.com/phnmnl/container-openms	(Sturm *et al.*, 2008)
OpenSwathRTNormalizer	container-openms	Mass Spectrometry	https://github.com/phnmnl/container-openms	(Sturm *et al.*, 2008)
PeakPickerHiRes	container-openms	Mass Spectrometry	https://github.com/phnmnl/container-openms	(Sturm *et al.*, 2008)

PeakPickerWavelet	container-openms	Mass Spectrometry	https://github.com/phnmnl/container-openms	(Sturm *et al.*, 2008)
PepNovoAdapter	container-openms	Mass Spectrometry	https://github.com/phnmnl/container-openms	(Sturm *et al.*, 2008)
PeptideIndexer	container-openms	Mass Spectrometry	https://github.com/phnmnl/container-openms	(Sturm *et al.*, 2008)
PhosphoScoring	container-openms	Mass Spectrometry	https://github.com/phnmnl/container-openms	(Sturm *et al.*, 2008)
PrecursorIonSelector	container-openms	Mass Spectrometry	https://github.com/phnmnl/container-openms	(Sturm *et al.*, 2008)
PrecursorMassCorrector	container-openms	Mass Spectrometry	https://github.com/phnmnl/container-openms	(Sturm *et al.*, 2008)
ProteinInference	container-openms	Mass Spectrometry	https://github.com/phnmnl/container-openms	(Sturm *et al.*, 2008)
ProteinQuantifier	container-openms	Mass Spectrometry	https://github.com/phnmnl/container-openms	(Sturm *et al.*, 2008)
ProteinResolver	container-openms	Mass Spectrometry	https://github.com/phnmnl/container-openms	(Sturm *et al.*, 2008)
PTModel	container-openms	Mass Spectrometry	https://github.com/phnmnl/container-openms	(Sturm *et al.*, 2008)
PTPredict	container-openms	Mass Spectrometry	https://github.com/phnmnl/container-openms	(Sturm *et al.*, 2008)
RTModel	container-openms	Mass Spectrometry	https://github.com/phnmnl/container-openms	(Sturm *et al.*, 2008)
RTPredict	container-openms	Mass Spectrometry	https://github.com/phnmnl/container-openms	(Sturm *et al.*, 2008)
SeedListGenerator	container-openms	Mass Spectrometry	https://github.com/phnmnl/container-openms	(Sturm *et al.*, 2008)
SpecLibSearcher	container-openms	Mass Spectrometry	https://github.com/phnmnl/container-openms	(Sturm *et al.*, 2008)
SpectraFilterBernNorm	container-openms	Mass Spectrometry	https://github.com/phnmnl/container-openms	(Sturm *et al.*, 2008)
SpectraFilterMarkerMower	container-openms	Mass Spectrometry	https://github.com/phnmnl/container-openms	(Sturm *et al.*, 2008)
SpectraFilterNLargest	container-openms	Mass Spectrometry	https://github.com/phnmnl/container-openms	(Sturm *et al.*, 2008)
SpectraFilterNormalizer	container-openms	Mass Spectrometry	https://github.com/phnmnl/container-openms	(Sturm *et al.*, 2008)

SpectraFilterParentPeakMower	container-openms	Mass Spectrometry	https://github.com/phnmnl/container-openms	(Sturm *et al.*, 2008)
SpectraFilterScaler	container-openms	Mass Spectrometry	https://github.com/phnmnl/container-openms	(Sturm *et al.*, 2008)
SpectraFilterSqrtMower	container-openms	Mass Spectrometry	https://github.com/phnmnl/container-openms	(Sturm *et al.*, 2008)
SpectraFilterThresholdMower	container-openms	Mass Spectrometry	https://github.com/phnmnl/container-openms	(Sturm *et al.*, 2008)
SpectraFilterWindowMower	container-openms	Mass Spectrometry	https://github.com/phnmnl/container-openms	(Sturm *et al.*, 2008)
SpectraMerger	container-openms	Mass Spectrometry	https://github.com/phnmnl/container-openms	(Sturm *et al.*, 2008)
TextExporter	container-openms	Mass Spectrometry	https://github.com/phnmnl/container-openms	(Sturm *et al.*, 2008)
TMTAnalyzer	container-openms	Mass Spectrometry	https://github.com/phnmnl/container-openms	(Sturm *et al.*, 2008)
TOFCalibration	container-openms	Mass Spectrometry	https://github.com/phnmnl/container-openms	(Sturm *et al.*, 2008)
XTandemAdapter	container-openms	Mass Spectrometry	https://github.com/phnmnl/container-openms	(Sturm *et al.*, 2008)
mtbls_nmr_raw_dummy_importer	container-nmrmlconv	Nuclear Magnetic Resonance	https://github.com/phnmnl/container-nmrmlconv	(Rocca-Serra *et al.*, 2016)
nmrmlconv	container-nmrmlconv	Nuclear Magnetic Resonance	https://github.com/phnmnl/container-nmrmlconv	(Rocca-Serra *et al.*, 2016)
zip-nmrml-collection	container-nmrmlconv	Nuclear Magnetic Resonance	https://github.com/phnmnl/container-nmrmlconv	(Rocca-Serra *et al.*, 2016)
metfrag-vis	container-metfrag-vis	Mass Spectrometry	https://github.com/phnmnl/container-metfrag-vis	Not Available
metfrag-vis-multiple	container-metfrag-vis	Mass Spectrometry	https://github.com/phnmnl/container-metfrag-vis	Not Available
ecomet_download	container-ecomet	Eco-metabolomics	https://github.com/phnmnl/container-ecomet	Not Available

ecomet_raw_extract	container-ecomet	Eco-metabolomics	https://github.com/phnmnl/container-ecomet	Not Available
ecomet_qc_extract	container-ecomet	Eco-metabolomics	https://github.com/phnmnl/container-ecomet	Not Available
ecomet_quality_control	container-ecomet	Eco-metabolomics	https://github.com/phnmnl/container-ecomet	Not Available
ecomet_preparations	container-ecomet	Eco-metabolomics	https://github.com/phnmnl/container-ecomet	Not Available
ecomet_import_maf	container-ecomet	Eco-metabolomics	https://github.com/phnmnl/container-ecomet	Not Available
ecomet_peak_picking	container-ecomet	Eco-metabolomics	https://github.com/phnmnl/container-ecomet	Not Available
ecomet_import_traits	container-ecomet	Eco-metabolomics	https://github.com/phnmnl/container-ecomet	Not Available
ecomet_species_diversity	container-ecomet	Eco-metabolomics	https://github.com/phnmnl/container-ecomet	Not Available
ecomet_species_shannon	container-ecomet	Eco-metabolomics	https://github.com/phnmnl/container-ecomet	Not Available
ecomet_species_unique	container-ecomet	Eco-metabolomics	https://github.com/phnmnl/container-ecomet	Not Available
ecomet_species_variability	container-ecomet	Eco-metabolomics	https://github.com/phnmnl/container-ecomet	Not Available
ecomet_concentration	container-ecomet	Eco-metabolomics	https://github.com/phnmnl/container-ecomet	Not Available
ecomet_species_features	container-ecomet	Eco-metabolomics	https://github.com/phnmnl/container-ecomet	Not Available
ecomet_species_venn	container-ecomet	Eco-metabolomics	https://github.com/phnmnl/container-ecomet	Not Available
ecomet_species_varpart	container-ecomet	Eco-metabolomics	https://github.com/phnmnl/container-ecomet	Not Available
ecomet_species_nmds	container-ecomet	Eco-metabolomics	https://github.com/phnmnl/container-ecomet	Not Available
ecomet_species_marchantia	container-ecomet	Eco-metabolomics	https://github.com/phnmnl/container-ecomet	Not Available
ecomet_ecology_varpart	container-ecomet	Eco-metabolomics	https://github.com/phnmnl/container-ecomet	Not Available
ecomet_ecology_rda	container-ecomet	Eco-metabolomics	https://github.com/phnmnl/container-ecomet	Not Available

ecomet_phylogeny	container-ecomet	Eco-metabolomics	https://github.com/phnmnl/container-ecomet	Not Available
ecomet_seasons_shannon	container-ecomet	Eco-metabolomics	https://github.com/phnmnl/container-ecomet	Not Available
ecomet_seasons_unique	container-ecomet	Eco-metabolomics	https://github.com/phnmnl/container-ecomet	Not Available
ecomet_seasons_variability	container-ecomet	Eco-metabolomics	https://github.com/phnmnl/container-ecomet	Not Available
ecomet_seasons_concentration	container-ecomet	Eco-metabolomics	https://github.com/phnmnl/container-ecomet	Not Available
ecomet_seasons_features	container-ecomet	Eco-metabolomics	https://github.com/phnmnl/container-ecomet	Not Available
ecomet_seasons_rda	container-ecomet	Eco-metabolomics	https://github.com/phnmnl/container-ecomet	Not Available
ecomet_seasons_nmds	container-ecomet	Eco-metabolomics	https://github.com/phnmnl/container-ecomet	Not Available
passatutto	container-passatutto	Mass Spectrometry	https://github.com/phnmnl/container-passatutto	(Scheubert *et al.*, 2017)
Metabolite_ID_Converter	container-metaboliteidconverter	Miscellaneous	https://github.com/phnmnl/container-metaboliteidconverter	(Wohlgemuth *et al.*, 2010)
Multivariate	container-multivariate	Statistics	https://github.com/phnmnl/container-multivariate	(Thevenot *et al.*, 2015)
isa_get_factors_summary	container-mtblisa	Data	https://github.com/phnmnl/container-mtblisa	(Sansone *et al.*, 2016; Haug *et al.*, 2013; Sansone *et al.*, 2012)
ISAslicer2	container-mtblisa	Data	https://github.com/phnmnl/container-mtblisa	(Sansone *et al.*, 2016; Haug *et al.*, 2013; Sansone *et al.*, 2012)
ISAslicer2_data_filter	container-mtblisa	Data	https://github.com/phnmnl/container-mtblisa	(Sansone *et al.*, 2016; Haug *et al.*, 2013; Sansone *et al.*, 2012)

metfrag-cli-batch	container-metfrag-cli-batch	Mass Spectrometry	https://github.com/phnmnl/container-metfrag-cli-batch	(Wolf *et al.*, 2010)
metfrag-cli-batch-multiple	container-metfrag-cli-batch	Mass Spectrometry	https://github.com/phnmnl/container-metfrag-cli-batch	(Ruttkies *et al.*, 2016)
ms-vfetc	container-ms-vfetc	Mzquality	https://github.com/phnmnl/container-ms-vfetc	Not Available
mzquality-rtshifts	container-mzquality	Mzquality	https://github.com/phnmnl/container-mzquality	Not Available
mzquality-blank-effect	container-mzquality	Mzquality	https://github.com/phnmnl/container-mzquality	Not Available
mzquality-qc-correction	container-mzquality	Mzquality	https://github.com/phnmnl/container-mzquality	Not Available
mzquality-qc-rsd	container-mzquality	Mzquality	https://github.com/phnmnl/container-mzquality	Not Available
mzquality-rep-rsd	container-mzquality	Mzquality	https://github.com/phnmnl/container-mzquality	Not Available
mzquality-is-rsd	container-mzquality	Mzquality	https://github.com/phnmnl/container-mzquality	Not Available
mzquality-export-measurements	container-mzquality	Mzquality	https://github.com/phnmnl/container-mzquality	Not Available
mzquality-plot-compounds	container-mzquality	Mzquality	https://github.com/phnmnl/container-mzquality	Not Available
rnmr1d	container-rnmr1d	Nuclear Magnetic Resonance	https://github.com/phnmnl/container-rnmr1d	Not Available
rnmr1d-stacked-plot	container-rnmr1d	Nuclear Magnetic Resonance	https://github.com/phnmnl/container-rnmr1d	Not Available
rnmr1d-prepare-output	container-rnmr1d	Nuclear Magnetic Resonance	https://github.com/phnmnl/container-rnmr1d	Not Available

nmr-integrals	container-nmr-integrals	Nuclear Magnetic Resonance	https://github.com/phnmnl/container-nmr-integrals	Not Available
metabomatching	container-metabomatching	Nuclear Magnetic Resonance	https://github.com/phnmnl/container-metabomatching	Not Available
iterative-signature-algorithm	container-iterative-signature-algorithm	Statistical Analysis Tools	https://github.com/phnmnl/container-iterative-signature-algorithm	(Bergmann *et al.*, 2003)
isatab2json	container-isatab2json	Data	https://github.com/phnmnl/container-isatab2json	(Sansone *et al.*, 2016, 2012)
batman-nmr	container-batman	Nuclear Magnetic Resonance	https://github.com/phnmnl/container-batman	(Hao *et al.*, 2014; Liebeke *et al.*, 2013; Hao *et al.*, 2012; Astle *et al.*, 2012)
nmrml2batman	container-nmrml2batman	Nuclear Magnetic Resonance	https://github.com/phnmnl/container-nmrml2batman	
isodyn	container-isodyn		https://github.com/phnmnl/container-isodyn	(Selivanov *et al.*, 2010; de Mas *et al.*, 2011; Selivanov *et al.*, 2005, 2004)
bruker2batman	container-bruker2batman	Nuclear Magnetic Resonance	https://github.com/phnmnl/container-bruker2batman	Not Available
metfragcli	container-metfrag-cli	Mass Spectrometry	https://github.com/phnmnl/container-metfrag-cli	(Ruttkies *et al.*, 2016)
mw2isatab	container-mw2isa	Data	https://github.com/phnmnl/container-mw2isa	(Sansone *et al.*, 2016; Sud *et al.*, 2016)
nmrml2isa	container-nmrml2isa	Data	https://github.com/phnmnl/container-nmrml2isa	(Rocca-Serra *et al.*, 2010)

msconvert2	container-pwiz	Mass Spectrometry	https://github.com/phnmnl/container-pwiz	(Chambers *et al.*, 2012)
isatab_validator	container-isatab-validator	Data	https://github.com/phnmnl/container-isatab-validator	(Sansone *et al.*, 2016, 2012)
metabolab	container-metabolab	Nuclear Magnetic Resonance	https://github.com/phnmnl/container-metabolab	(Ludwig and Günther, 2011)
lcmsmatching	container-lcmsmatching	Mass Spectrometry	https://github.com/phnmnl/container-lcmsmatching	Not Available
Univariate	container-univariate	Statistical Analysis	https://github.com/phnmnl/container-univariate	(Benjamini and Hochberg, 1995; Dalgaard, 2008; Pohlert, 2015)
mzml2isa	container-mzml2isa	Data	https://github.com/phnmnl/container-mzml2isa	(Rocca-Serra *et al.*, 2010)
xcms-find-peaks	container-xcms-1.x	Mass Spectrometry	https://github.com/phnmnl/container-xcms-1.x	(Benton *et al.*, 2008)
xcms-collect-peaks	container-xcms-1.x	Mass Spectrometry	https://github.com/phnmnl/container-xcms-1.x	(Benton *et al.*, 2008)
xcms-group-peaks	container-xcms-1.x	Mass Spectrometry	https://github.com/phnmnl/container-xcms-1.x	(Benton *et al.*, 2008)
xcms-correct-rt	container-xcms-1.x	Mass Spectrometry	https://github.com/phnmnl/container-xcms-1.x	(Benton *et al.*, 2008)
xcms-fill-peaks	container-xcms-1.x	Mass Spectrometry	https://github.com/phnmnl/container-xcms-1.x	(Benton *et al.*, 2008)
xcmssplit	container-xcms-1.x	Mass Spectrometry	https://github.com/phnmnl/container-xcms-1.x	(Benton *et al.*, 2008)

xcms-blankfilter	container-xcms-1.x	Mass Spectrometry	https://github.com/phnmnl/container-xcms-1.x	(Benton *et al.*, 2008)
xcms-dilutionfilter	container-xcms-1.x	Mass Spectrometry	https://github.com/phnmnl/container-xcms-1.x	(Benton *et al.*, 2008)
save_chromatogram	container-xcms	Mass Spectrometry	https://github.com/phnmnl/container-xcms	(Benton *et al.*, 2008)
show_chromatogram	container-xcms	Mass Spectrometry	https://github.com/phnmnl/container-xcms	(Benton *et al.*, 2008)
pathway_enrichment	container-pathwayenrichment	Post-processing	https://github.com/phnmnl/container-pathwayenrichment	(Thiele *et al.*, 2013; Cottret *et al.*, 2018)
fingerprint_clustering	container-fingerprintclustering	Post-processing	https://github.com/phnmnl/container-fingerprintclustering	(Cottret *et al.*, 2018; Rousseeuw, 1987; Tibshirani *et al.*, 2001)
fingerprint_subnetwork	container-fingerprintsubnetwork	Post-processing	https://github.com/phnmnl/container-fingerprintsubnetwork	(Thiele *et al.*, 2013; Cottret *et al.*, 2018)
mtbls-downloader	container-scp-aspera		https://github.com/phnmnl/container-scp-aspera	(Haug *et al.*, 2013)
consensusXMLToXcms	container-camera	Mass Spectrometry	https://github.com/phnmnl/container-camera	(Kuhl *et al.*, 2012)
cameraToFeatureXML	container-camera	Mass Spectrometry	https://github.com/phnmnl/container-camera	(Kuhl *et al.*, 2012)
featureXMLToCAMERA	container-camera	Mass Spectrometry	https://github.com/phnmnl/container-camera	(Kuhl *et al.*, 2012)
featureXMLToXcms	container-camera	Mass Spectrometry	https://github.com/phnmnl/container-camera	(Kuhl *et al.*, 2012)
zip-collection	container-camera	Mass Spectrometry	https://github.com/phnmnl/container-camera	(Kuhl *et al.*, 2012)
unzip-collection	container-camera	Mass Spectrometry	https://github.com/phnmnl/container-camera	(Kuhl *et al.*, 2012)
camera-annotate-peaks	container-camera	Mass Spectrometry	https://github.com/phnmnl/container-camera	(Kuhl *et al.*, 2012)
camera-group-fwhm	container-camera	Mass Spectrometry	https://github.com/phnmnl/container-camera	(Kuhl *et al.*, 2012)
camera-group-corr	container-camera	Mass Spectrometry	https://github.com/phnmnl/container-camera	(Kuhl *et al.*, 2012)

camera-find-isotopes	container-camera	Mass Spectrometry	https://github.com/phnmnl/container-camera	(Kuhl *et al.*, 2012)
camera-find-adducts	container-camera	Mass Spectrometry	https://github.com/phnmnl/container-camera	(Kuhl *et al.*, 2012)
camera-prepareoutput	container-camera	Mass Spectrometry	https://github.com/phnmnl/container-camera	(Kuhl *et al.*, 2012)
msnbase-read-msms	container-msnbase	Mass Spectrometry	https://github.com/phnmnl/container-msnbase	(Gatto and Lilley, 2012)
map-msms2camera	container-msnbase	Mass Spectrometry	https://github.com/phnmnl/container-msnbase	(Gatto and Lilley, 2012)
msms2metfrag	container-msnbase	Mass Spectrometry	https://github.com/phnmnl/container-msnbase	(Gatto and Lilley, 2012)
msms2metfrag-multiple	container-msnbase	Mass Spectrometry	https://github.com/phnmnl/container-msnbase	(Gatto and Lilley, 2012)
msnbase-filter-merge-msms	container-msnbase	Mass Spectrometry	https://github.com/phnmnl/container-msnbase	(Gatto and Lilley, 2012)
aggregatemetfrag	container-msnbase	Mass Spectrometry	https://github.com/phnmnl/container-msnbase	(Gatto and Lilley, 2012)
mtbls-labs-uploader	container-mtbl-labs-uploader	Data	https://github.com/phnmnl/container-mtbl-labs-uploader	(Haug *et al.*, 2013)
isacreate_metabo	container-isatab-create	Data	https://github.com/phnmnl/container-isatab-create	(Haug *et al.*, 2013; Sansone *et al.*, 2016, 2012)
dimspy_process_scans	container-dimspy	Mass Spectrometry	https://github.com/phnmnl/container-dimspy	(Southam *et al.*, 2016; Kirwan *et al.*, 2014; Southam *et al.*, 2007; Weber *et al.*, 2011)
dimspy_replicate_filter	container-dimspy	Mass Spectrometry	https://github.com/phnmnl/container-dimspy	(Southam *et al.*, 2016; Kirwan *et al.*, 2014; Southam *et al.*, 2007; Weber *et al.*, 2011)

dimspy_align_samples	container-dimspy	Mass Spectrometry	https://github.com/phnmnl/container-dimspy	(Southam *et al.*, 2016; Kirwan *et al.*, 2014; Southam *et al.*, 2007; Weber *et al.*, 2011)
dimspy_blank_filter	container-dimspy	Mass Spectrometry	https://github.com/phnmnl/container-dimspy	(Southam *et al.*, 2016; Kirwan *et al.*, 2014; Southam *et al.*, 2007; Weber *et al.*, 2011)
dimspy_sample_filter	container-dimspy	Mass Spectrometry	https://github.com/phnmnl/container-dimspy	(Southam *et al.*, 2016; Kirwan *et al.*, 2014; Southam *et al.*, 2007; Weber *et al.*, 2011)
dimspy_hdf5_to_txt	container-dimspy	Mass Spectrometry	https://github.com/phnmnl/container-dimspy	(Southam *et al.*, 2016; Kirwan *et al.*, 2014; Southam *et al.*, 2007; Weber *et al.*, 2011)
dimspy_merge_peaklists	container-dimspy	Mass Spectrometry	https://github.com/phnmnl/container-dimspy	(Southam *et al.*, 2016; Kirwan *et al.*, 2014; Southam *et al.*, 2007; Weber *et al.*, 2011)
dimspy_get_peaklists	container-dimspy	Mass Spectrometry	https://github.com/phnmnl/container-dimspy	(Southam *et al.*, 2016; Kirwan *et al.*, 2014; Southam *et al.*, 2007; Weber *et al.*, 2011)
melt	container-reshape2-melt	Statistical Analysis	https://github.com/phnmnl/container-reshape2-melt	Not Available
cast	container-reshape2-cast	Statistical Analysis	https://github.com/phnmnl/container-reshape2-cast	Not Available

Astle,W. *et al.* (2012) A Bayesian Model of NMR Spectra for the Deconvolution and Quantification of Metabolites in Complex Biological Mixtures. *J. Am. Stat. Assoc.*, **107**, 1259–1271.

Benjamini,Y. and Hochberg,Y. (1995) Controlling the False Discovery Rate: A Practical and Powerful Approach to Multiple Testing. *J. R. Stat. Soc. Series B Stat. Methodol.*, **57**, 289–300.

Benton,H.P. *et al.* (2008) XCMS2: processing tandem mass spectrometry data for metabolite identification and structural characterization. *Anal. Chem.*, **80**, 6382–6389.

Bergmann,S. *et al.* (2003) Iterative signature algorithm for the analysis of large-scale gene expression data. *Phys. Rev. E Stat. Nonlin. Soft Matter Phys.*, **67**, 031902.

Blaise,B.J. *et al.* (2016) Power Analysis and Sample Size Determination in Metabolic Phenotyping. *Anal. Chem.*, **88**, 5179–5188.

Chambers,M.C. *et al.* (2012) A cross-platform toolkit for mass spectrometry and proteomics. In, *Nat Biotechnol*. United States, pp. 918–920.

Cottret,L. *et al.* (2018) MetExplore: collaborative edition and exploration of metabolic networks. *Nucleic Acids Res.*, **46**,

W495–W502.
Dalgaard,P. (2008) Introductory Statistics with R Springer Science & Business Media.
Dieterle,F. *et al.* (2006) Probabilistic quotient normalization as robust method to account for dilution of complex biological mixtures. Application in 1H NMR metabonomics. *Anal. Chem.*, **78**, 4281–4290.
Dunn,W.B. *et al.* (2011) Procedures for large-scale metabolic profiling of serum and plasma using gas chromatography and liquid chromatography coupled to mass spectrometry. *Nat. Protoc.*, **6**, 1060–1083.
Gatto,L. and Lilley,K.S. (2012) MSnbase-an R/Bioconductor package for isobaric tagged mass spectrometry data visualization, processing and quantitation. *Bioinformatics*, **28**, 288–289.
Giacomoni,F. *et al.* (2015) Workflow4Metabolomics: a collaborative research infrastructure for computational metabolomics. *Bioinformatics*, **31**, 1493–1495.
González-Beltrán,A. *et al.* (2014) The Risa R/Bioconductor package: integrative data analysis from experimental metadata and back again. *BMC Bioinformatics*, **15 Suppl 1**, S11.
Hao,J. *et al.* (2012) BATMAN--an R package for the automated quantification of metabolites from nuclear magnetic resonance spectra using a Bayesian model. *Bioinformatics*, **28**, 2088–2090.
Hao,J. *et al.* (2014) Bayesian deconvolution and quantification of metabolites in complex 1D NMR spectra using BATMAN. *Nat. Protoc.*, **9**, 1416–1427.
Haug,K. *et al.* (2013) MetaboLights—an open-access general-purpose repository for metabolomics studies and associated meta-data. *Nucleic Acids Res.*, **41**, D781–D786.
Kirwan,J.A. *et al.* (2014) Direct infusion mass spectrometry metabolomics dataset: a benchmark for data processing and quality control. *Sci Data*, **1**, 140012.
van der Kloet,F.M. *et al.* (2009) Analytical error reduction using single point calibration for accurate and precise metabolomic phenotyping. *J. Proteome Res.*, **8**, 5132–5141.
Kuhl,C. *et al.* (2012) CAMERA: an integrated strategy for compound spectra extraction and annotation of liquid chromatography/mass spectrometry data sets. *Anal. Chem.*, **84**, 283–289.
Liebeke,M. *et al.* (2013) Combining spectral ordering with peak fitting for one-dimensional NMR quantitative metabolomics. *Anal. Chem.*, **85**, 4605–4612.
Ludwig,C. and Günther,U.L. (2011) MetaboLab--advanced NMR data processing and analysis for metabolomics. *BMC Bioinformatics*, **12**, 366.
de Mas,I.M. *et al.* (2011) Compartmentation of glycogen metabolism revealed from 13C isotopologue distributions. *BMC Syst. Biol.*, **5**, 175.
Mason,R.L. *et al.* (1997) A Practical Approach for Interpreting Multivariate T2 Control Chart Signals. *J. Commod. Sci. Technol. Qual.*, **29**, 396–406.
Pohlert,T. (2015) PMCMR: Calculate pairwise multiple comparisons of mean rank sums. *R package version*, **1**.
Rinaudo,P. *et al.* (2016) biosigner: A New Method for the Discovery of Significant Molecular Signatures from Omics Data. *Front Mol Biosci*, **3**, 26.
Rocca-Serra,P. *et al.* (2016) Data standards can boost metabolomics research, and if there is a will, there is a way. *Metabolomics*, **12**, 14.
Rocca-Serra,P. *et al.* (2010) ISA software suite: supporting standards-compliant experimental annotation and enabling curation at the community level. *Bioinformatics*, **26**, 2354–2356.
Rousseeuw,P.J. (1987) Silhouettes: A graphical aid to the interpretation and validation of cluster analysis. *J. Comput. Appl. Math.*, **20**, 53–65.
Ruttkies,C. *et al.* (2016) MetFrag relaunched: incorporating strategies beyond in silico fragmentation. *J. Cheminform.*, **8**, 3.
Sansone,S.-A. *et al.* (2016) ISA Model and Serialization Specifications 1.0.
Sansone,S.-A. *et al.* (2012) Toward interoperable bioscience data. *Nat. Genet.*, **44**, 121–126.
Scheubert,K. *et al.* (2017) Significance estimation for large scale metabolomics annotations by spectral matching. *Nat. Commun.*, **8**, 1494.
Selivanov,V.A. *et al.* (2004) An optimized algorithm for flux estimation from isotopomer distribution in glucose metabolites. *Bioinformatics*, **20**, 3387–3397.
Selivanov,V.A. *et al.* (2010) Edelfosine-induced metabolic changes in cancer cells that precede the overproduction of reactive oxygen species and apoptosis. *BMC Syst. Biol.*, **4**, 135.
Selivanov,V.A. *et al.* (2017) MIDcor, an R-program for deciphering mass interferences in mass spectra of metabolites enriched in stable isotopes. *BMC Bioinformatics*, **18**, 88.
Selivanov,V.A. *et al.* (2005) Rapid simulation and analysis of isotopomer distributions using constraints based on enzyme

mechanisms: an example from HT29 cancer cells. *Bioinformatics*, **21**, 3558–3564.
Southam,A.D. *et al.* (2016) A complete workflow for high-resolution spectral-stitching nanoelectrospray direct-infusion mass-spectrometry-based metabolomics and lipidomics. *Nat. Protoc.*, **12**, 310–328.
Southam,A.D. *et al.* (2007) Dynamic range and mass accuracy of wide-scan direct infusion nanoelectrospray Fourier transform ion cyclotron resonance mass spectrometry-based metabolomics increased by the spectral stitching method. *Anal. Chem.*, **79**, 4595–4602.
Sturm,M. *et al.* (2008) OpenMS - an open-source software framework for mass spectrometry. *BMC Bioinformatics*, **9**, 163.
Sud,M. *et al.* (2016) Metabolomics Workbench: An international repository for metabolomics data and metadata, metabolite standards, protocols, tutorials and training, and analysis tools. *Nucleic Acids Res.*, **44**, D463–70.
Thevenot,E.A. *et al.* (2015) Analysis of the Human Adult Urinary Metabolome Variations with Age, Body Mass Index, and Gender by Implementing a Comprehensive Workflow for Univariate and OPLS Statistical Analyses. *J. Proteome Res.*, **14**, 3322–3335.
Thiele,I. *et al.* (2013) A community-driven global reconstruction of human metabolism. *Nat. Biotechnol.*, **31**, 419–425.
Tibshirani,R. *et al.* (2001) Estimating the number of clusters in a data set via the gap statistic. *J. R. Stat. Soc. Series B Stat. Methodol.*, **63**, 411–423.
Veselkov,K.A. *et al.* (2011) Optimized preprocessing of ultra-performance liquid chromatography/mass spectrometry urinary metabolic profiles for improved information recovery. *Anal. Chem.*, **83**, 5864–5872.
Weber,R.J.M. *et al.* (2011) Characterization of isotopic abundance measurements in high resolution FT-ICR and Orbitrap mass spectra for improved confidence of metabolite identification. *Anal. Chem.*, **83**, 3737–3743.
Wohlgemuth,G. *et al.* (2010) The Chemical Translation Service—a web-based tool to improve standardization of metabolomic reports. *Bioinformatics*, **26**, 2647–2648.
Wolf,S. *et al.* (2010) In silico fragmentation for computer assisted identification of metabolite mass spectra. *BMC Bioinformatics*, **11**, 148.
